# Discovery of Novel Pyridazine-Based Cyclooxygenase-2 Inhibitors with a Promising Gastric Safety Profile

**DOI:** 10.3390/molecules25092002

**Published:** 2020-04-25

**Authors:** Abida Khan, Anupama Diwan, Hamdy Kh. Thabet, Mohd Imran, Md. Afroz Bakht

**Affiliations:** 1 School of Pharmaceutical Sciences, Apeejay Stya University, Sohna - Palwal Road, Sohna 122103, India; anupama.diwan@asu.apeejay.edu; 2 Department of Chemistry, Faculty of Arts and Science, Northern Border University, Rafha 91911, Saudi Arabia; hamdykhamees@gmail.com; 3 Department of Pharmaceutical Chemistry, Faculty of Pharmacy, Northern Border University, Rafha 91911, Saudi Arabia; imran.pchem@gmail.com; 4 Department of Chemistry, College of Science and Humanities, Prince Sattam Bin Abdulaziz University, Al-Kharj 11942, Saudi Arabia; m.bakht@psau.edu.sa

**Keywords:** pyridazine, thiazole, 4-thiazolidinone, cyclooxygenase-2, non-ulcerogenic, lipid peroxidation, Lipinski’s rule

## Abstract

Cyclooxygenase-2 (COX-2) is implicated in the development of chronic inflammatory diseases. Recently, pyridazine derivatives have emerged as a novel prototype to develop COX-2 inhibitors. Accordingly, some pyridazine-based COX-2 inhibitors are reported herein. The reaction of aldehyde **3** and different hydrazines yielded the corresponding hydrazones. The hydrazones were further derivatized to the title compounds, which were assessed for COX-1 and COX-2 inhibitory action, gastric ulcerogenic effects, and lipid peroxidation properties. Molecular docking studies and determination of the physicochemical parameters were also carried out. The allocated structures of the reported compounds were coherent with their spectroscopic data. The compounds **9a** (IC_50_ = 15.50 nM, 114.77%), **9b** (IC_50_ = 17.50 nM, 101.65%), **12** (IC_50_ = 17.10 nM, 104.03%), **16b** (IC_50_ = 16.90 nM, 105.26%), and **17** (IC_50_ = 17.70 nM, 100.5%) displayed better COX-2 inhibition than celecoxib (IC_50_ = 17.79 nM, 100%). These outcomes were harmonious with the molecular docking studies of **9a**, **9b**, **12**, **16b**, and **17**. These compounds also displayed comparable onset and the duration of action concerning celecoxib and indomethacin in the in vivo studies. No ulcerogenic effects were observed for **9a** and **12**, whereas **9b**, **16b**, and **17** showed an insignificant ulcerogenic effect compared to celecoxib. The compounds **9a**, **9b**, **12**, **16b**, and **17** displayed a better lipid peroxidation profile than celecoxib and indomethacin. The compounds **9a** (%ABS = 84.09), **9b** (%ABS = 84.09), **12** (%ABS = 66.87), **16b** (%ABS = 75.02), and **17** (%ABS = 81.42) also displayed appreciable calculated absorption compared to celecoxib (%ABS = 82.09). The compounds **9a**, **9b**, **11**, **16b**, and **17** have been recognized and postulated as non-ulcerogenic COX-2 inhibitors with promising physicochemical parameters and gastric safety profile. These compounds may be useful candidates to combat diseases caused by higher levels of COX-2.

## 1. Introduction

Inflammation is a self-protective reaction of human body tissues towards injurious stimuli like infection, irritants, poisonous substances, irradiation, and tissue injury [1]. The inflammation can be divided into acute inflammation and chronic inflammation. The symptoms of acute inflammation comprise of swelling, heat, immobility, redness, and pain, which may last for several days [2]. However, persistent inflammation for a longer time or chronic inflammation may lead to the development of diseases like gout, ankylosing spondylitis, osteoarthritis, rheumatoid arthritis, Alzheimer’s disease, ulcerative colitis, depression, epilepsy, irritable bowel diseases, kidney injury, cancer, asthma, hepatitis, pancreatitis, and atherosclerosis [1,3]. According to the published data, 40% and 61% of the population of the age > 60 years in the UK and Saudi Arabia, respectively, are suffering from arthritis [4]. It is estimated that about 1/4th of the adults in the USA will be affected by osteoarthritis by 2030 [5]. Non-steroidal anti-inflammatory drugs (NSAIDs) are regularly prescribed for chronic inflammatory diseases. NSAIDs inhibit the cyclooxygenase enzyme (COX). COX is accountable for transforming arachidonic acid into prostaglandins, which initiate the inflammatory events in a cell [1,2]. The constitutive COX-1 is responsible for the maintenance functions of the cell, including the safety of the gastric mucosa, aggregation of platelets, and control of the renal blood flow [6,7]. COX-2 cannot be detected in healthy cells. However, this inducible enzyme is produced intracellularly after harmful stimuli. It is responsible for the development of inflammatory events in a cell, which ultimately lead to the development of inflammatory diseases [1,3]. The commonly used NSAIDs cause ulcerogenic effects after prolonged use because they inhibit both COX-2 and COX-1 [6,7]. The ulcerogenic effect of NSAIDs is credited to the inhibition of COX-1, and the anti-inflammatory action is credited to the inhibition of COX-2 [8,9]. Based on this understanding, celecoxib, rofecoxib, and etoricoxib were developed as specific COX-2 inhibitors [10,11,12]. However, cerebrovascular risk and cardiac toxicity have been reported as adverse effects of some particular COX-2 inhibitors at the standard dose, for example, rofecoxib [12,13]. Accordingly, medicinal chemists are looking forward to developing new anti-inflammatory agents, which lack the aforementioned adverse effects and have a promising gastric safety profile [14].

Pyridazine, a famous diazine ring, is part of many pharmacodynamic agents like indolidan (antihypertensive), bemoradan (antihypertensive), levosimendan (congestive heart failure), pimobendan (congestive heart failure), milrinone (cardiotonic), minaprine (antidepressant), imazodan (PDE3 inhibitor), zardaverine (PDE3 inhibitor), and olaparib (anticancer) [15,16,17,18,19]. Emorfazone (Pentoil), an analgesic and anti-inflammatory pyridazine derivative, is in clinical use in Japan and is claimed to lack gastric side effects [19]. Similarly, Zomipirac has been derivatized to a pyridazine derivative, which had a COX-1/COX-2 selectivity index > 1500 [20,21]. Novel targets for developing anti-inflammatory agents bearing the pyridazine scaffold along with the general chemistry, mechanism of action, and the structure–activity relationship (SAR) have also been reported [19]. Ulcerogenic effects or gastrointestinal bleeding are the most frequently encountered adverse effects of the clinically used NSAIDs [13]. As per the recent publications, the pyridazine moiety is a new template for developing COX-2 inhibitors [15,16,17,18,19]. The possibility of the various structural modifications in the pyridazine ring, and the literature revealing the COX-2 inhibitory potential of the pyridazine nucleus [15,16,17,18,19,20,21] makes it a promising scaffold to develop non-ulcerogenic COX-2 inhibitors. As per the published reports, incorporation of the thiazole moiety and the 4-thiazolidinone moiety in a compound imparts selectivity towards COX-2 inhibition and also improves its gastric safety profile [22,23,24,25,26,27,28]. Accordingly, as an extension of our work to develop superior anti-inflammatory compounds [29,30,31,32], we report herein novel pyridazine-based thiazole and 4-thiazolidinone derivatives as cyclooxygenase-2 inhibitors with an improved gastric safety profile.

## 2. Results and Discussion

### 2.1. Chemistry

**Scheme 1:**
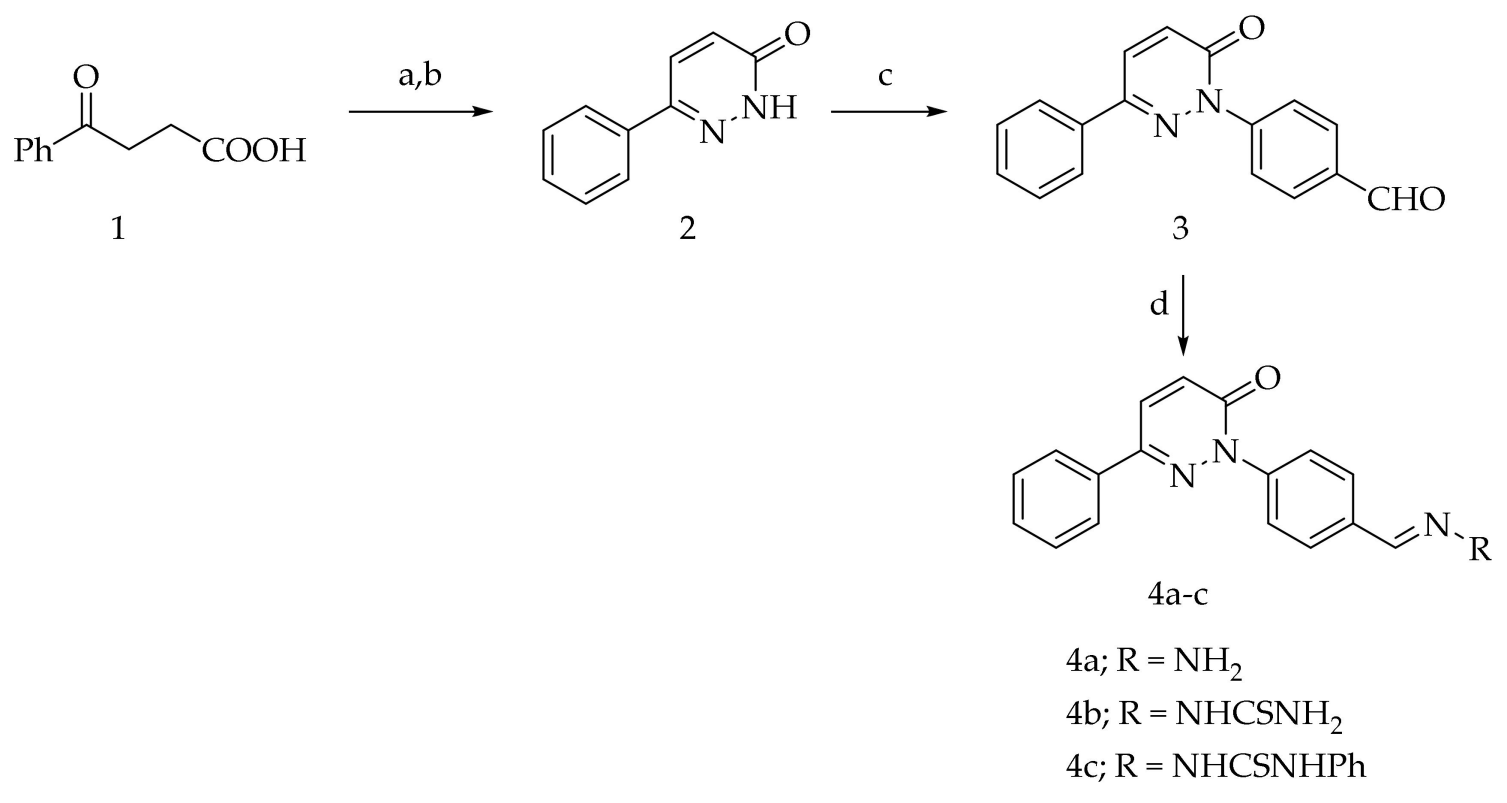
Preparation of **4a**–**c** (Reagents and conditions: (a) NH_2_NH_2_·H_2_O, EtOH, reflux; (b) Br_2_/AcOH, (c) 4-fluorobenzaldehyde, DMSO/K_2_CO_3_, reflux; (d) RNH_2_, EtOH, few drops AcOH, reflux).

The intermediates **2**, **3**, **4b**, and **4c** were reported in our previous publication [30]. The intermediates **1**, **5a**, **5b**, and **13** are available commercially. The condensation of aldehyde **3** with hydrazine hydrate, thiosemicarbazide, and phenylthiosemicarbazide afforded hydrazone **4a** and the thiosemicarbazone derivatives **4b**,**c**, respectively (Scheme 1).

**Scheme 2:**
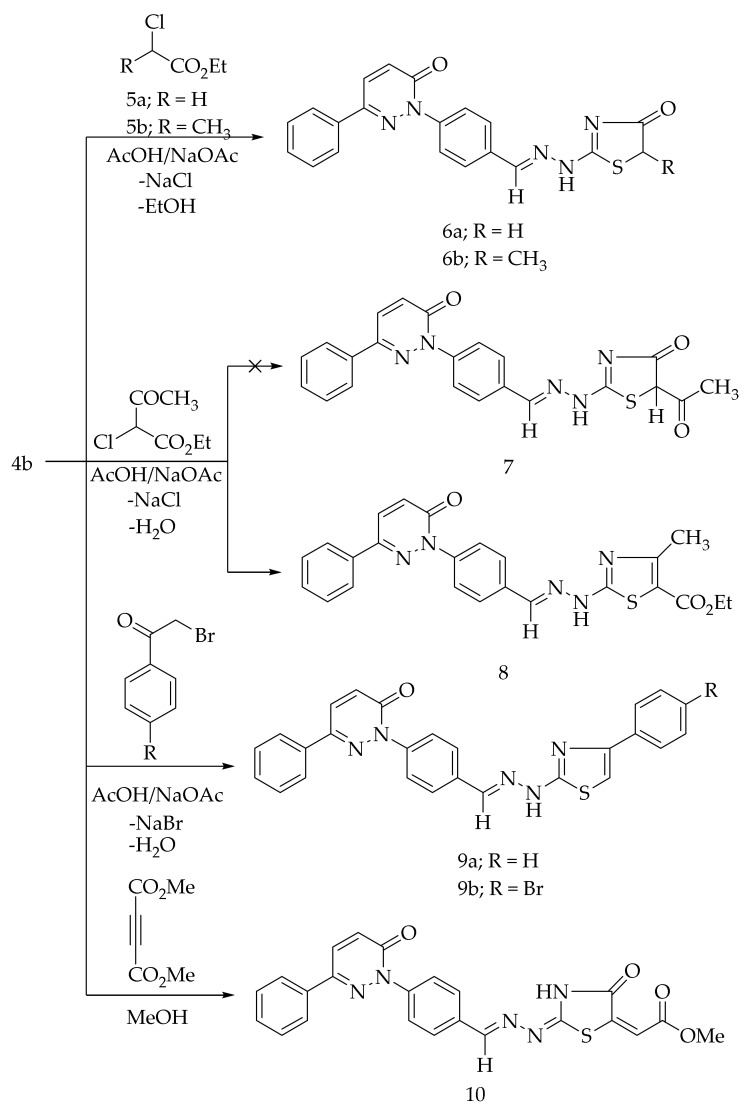
Preparation of **6a**,**b**, **8**, **9a**,**b**, and **10**.

The cycloalkylation of the thiocarbamoyl group of **4b** with the ethyl α-chloroacetate (**5a**) and ethyl α-chloropropionate (**5b**) in glacial acetic acid comprising a catalytic amount of the fused sodium acetate at the reflux temperature afforded the corresponding 4-thiazolidinone derivatives, **6a** and **6b**, respectively (Scheme 2). The formation of **6a** and **6b** is expected to proceed through the initial *S*-alkylation via the loss of sodium chloride followed by the intramolecular cyclization with the elimination of ethanol. The Hantzsch reaction of **4b** with the ethyl α-chloro acetoacetate in acetic acid in the presence of sodium acetate led to the formation of 4-methyl-thiazole derivative **8** (Scheme 2). The possibility of compound **7** was excluded based on the spectral analyses. The treatment of **4b** with 4-substituted phenacyl bromides in refluxing ethanol in the presence of anhydrous sodium acetate produced the corresponding thiazole derivatives, **9a** and **9b** (Scheme 2). The cyclization reaction of **4b** with dimethyl acetylenedicarboxylate in methanol provided the 4-thiazolidinone derivative **10** (Scheme 2).

**Scheme 3:**
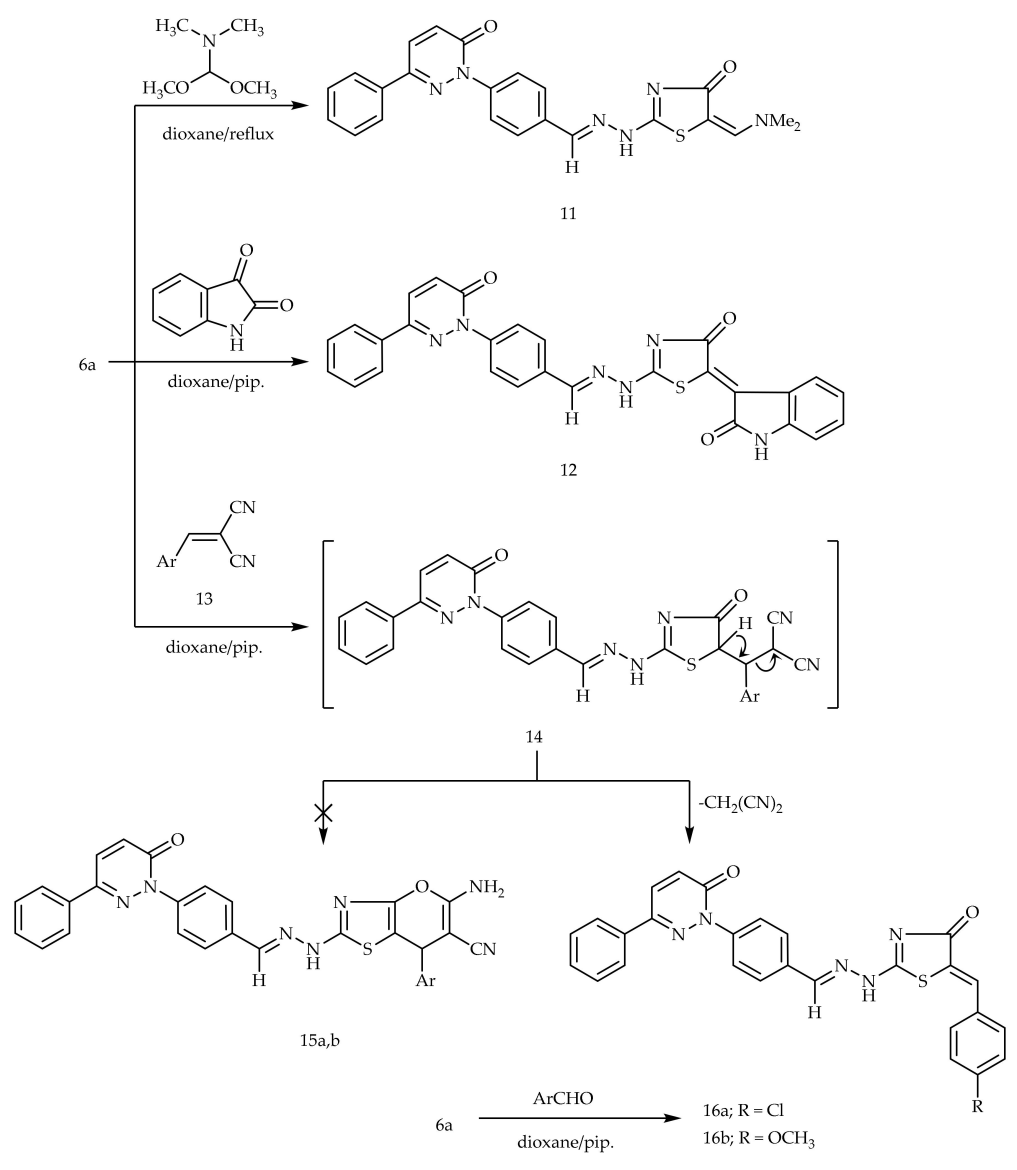
Preparation of **11**, **12**, and **16a**,**b**.

The condensation of the active methylene group of **6a** with electrophilic dimethylformamide-dimethylacetal (DMF-DMA) in dry dioxane afforded 5-dimethylaminomethylidine derivative **11** (Scheme 3). Similarly, the treatment of **6a** with isatin in dioxane comprising a catalytic amount of piperidine provided compound **12** (Scheme 3). The treatment of **6a** with α-cinnamonitriles in dioxane containing a catalytic amount of piperidine furnished the benzylidene derivatives **16a**,**b**, wherein other possible structures **15a**,**b** were ruled out based on the spectral data. Another synthetic route of compounds **16a**,**b** was achieved via the Knoevenagel condensation of compounds **6a** with the corresponding aromatic aldehydes (Scheme 3). 

**Scheme 4:**
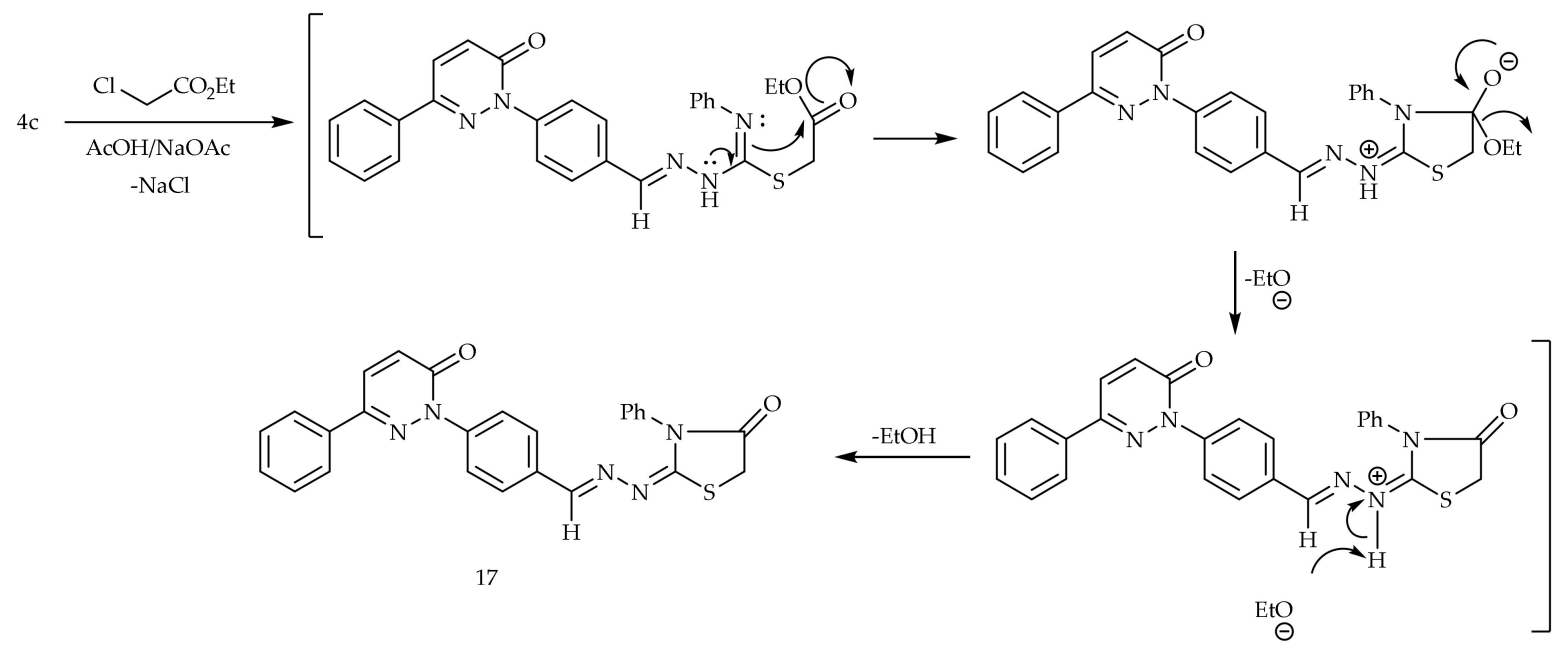
Preparation of **17**.

The cyclocondensation of the thiocarbamoyl group of **4c** with the ethyl α-chloroacetate in acetic acid containing a catalytic amount of the anhydrous sodium acetate provided 4-thiazolidinone derivative **17** (Scheme 4).

**Scheme 5:**
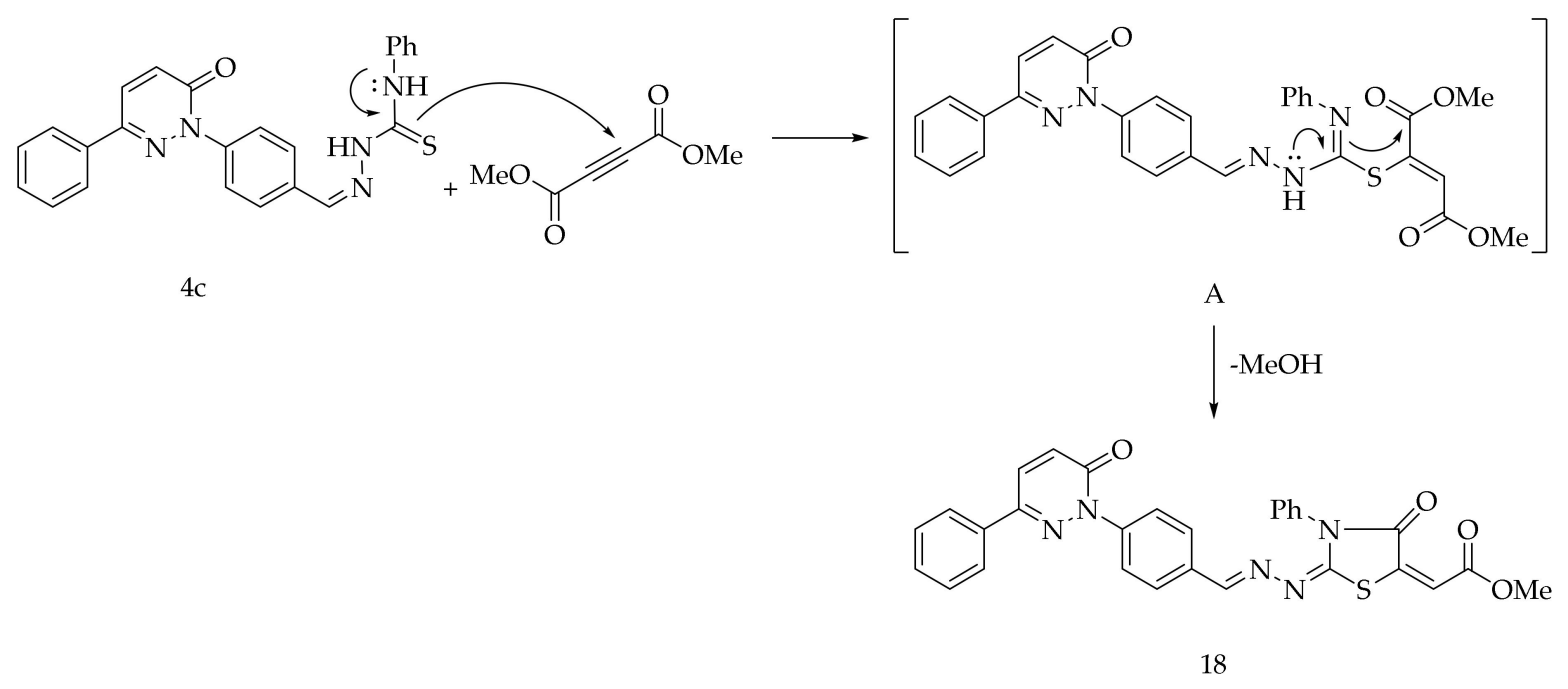
Preparation of **18**.

Finally, the reaction of **4c** with dimethyl acetylenedicarboxylate in methanol at the reflux temperature afforded the compound **18** (Scheme 5). The formation of **18** is expected to proceed via the nucleophilic addition of *S*-atom to the *sp*-carbon of the dimethyl acetylenedicarboxylate to generate the intermediate A. The intermediate A after the intramolecular cyclization through the nucleophilic amino group of the thiosemicarbazone yielded **18**.

There is a possibility of at least four or more geometrical isomers for some of the compounds, for example, **10**, **11**, **12**, **16a**, **16b**, **17**, and **18**. However, these isomers may be able to interconvert. Therefore, we have not mentioned the *E* or *Z* configuration in the nomenclature of our compounds. The structures of **4a, 6a, 6b, 8, 9a, 9b, 10, 11, 12, 16a, 16b, 17,** and **18** were proven on the basis of their spectroscopical data. The detailed spectroscopical data are provided in the experimental part.

### 2.2. Biological Activity

#### 2.2.1. In Vitro COX Inhibitory Action

The compounds **4a, 6a, 6b, 8, 9a, 9b, 10, 11, 12, 16a, 16b, 17,** and **18** were examined as COX-1 and COX-2 inhibitors along with indomethacin and celecoxib. It was performed by the 10-fold dilution technique utilizing test packs of the human COX-1/COX-2 (Cayman Chemicals, 560131, Ann Arbor, MI, USA) [30]. Indomethacin and celecoxib were used as standard drugs. Indomethacin is an ulcerogenic non-specific COX-1 and COX-2 inhibitor, whereas celecoxib is considered as a non-ulcerogenic-specific COX-2 inhibitor [6,10]. 

**Figure 1:**
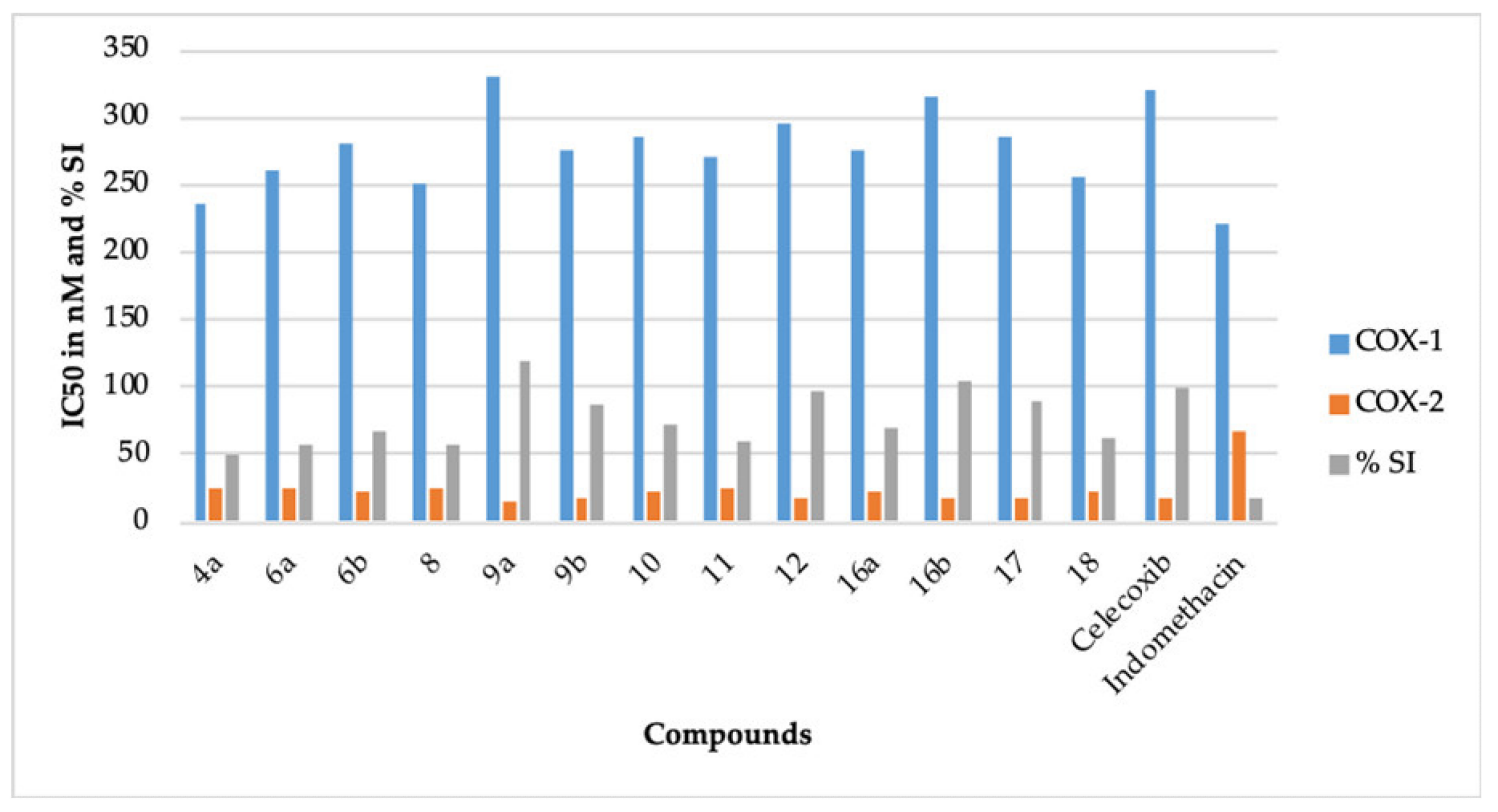
The IC_50_ (nM) and the % selectivity index (SI) of compounds **4a**, **6a**, **6b**, **8**, **9a**, **9b**, **10**, **11**, **12**, **16a**, **16b**, **17**, **18**, celecoxib, and indomethacin.

It is a well known fact that the inhibition of COX-1 is mainly responsible for the ulcerogenic effect of NSAIDs like indomethacin [6,7]. It is also well documented that specific COX-2 inhibitors like celecoxib are potent anti-inflammatory agents and possess a better gastric safety profile because they do not inhibit COX-1 [10]. Therefore, for a better comparison, the %COX-1 inhibition of indomethacin was normalized to 100% for COX-1, and the %COX-2 inhibition of celecoxib was normalized to 100% for COX-2 (Table 1). The selectivity index of celecoxib was also normalized to 100%. All the compounds comprising celecoxib (IC_50_ = 320 nM) displayed greater IC_50_ against COX-1, when compared to indomethacin (IC_50_ = 220 nM) (Table 1, Figure 1). This result points out that our compounds should have a better gastric safety profile than indomethacin [8,9]. Our belief is further strengthened by the fact that our compounds showed better inhibition of COX-2 in contrast to COX-1. The compounds **9a** (IC_50_ = 15.50 nM, 114.77%), **9b** (IC_50_ = 17.50 nM, 101.65%), **12** (IC_50_ = 17.10 nM, 104.03%), **16b** (IC_50_ = 16.90 nM, 105.26%), and **17** (IC_50_ = 17.70 nM, 100.5%) demonstrated better COX-2 inhibition than celecoxib (IC_50_ = 17.79, 100%). The selectivity index (SI) of **9a** (SI = 21.29, 118.40%) and **16b** (SI = 18.63, 103.61%) was superior to celecoxib (SI = 17.98, 100%). The SI of **9b** (SI = 15.71, 87.37%), **12** (SI = 17.25, 95.93%), and **17** (SI = 16.10, 89.54%) was also comparable to celecoxib (SI = 17.98, 100%). Based on the data mentioned above, **9a**, **9b**, **12**, **16b**, and **17** were chosen for the in vivo anti-inflammatory activity.

The novel pyridazine derivatives can be categorized as thiazole derivatives (**8, 9a**, and **9b**) and 4-thiazolidinone derivatives (**6a**, **6b**, **10**, **11**, **12, 16a**, **16b**, **17**, and **18**). It is apparent from Table 1 that the thiazole derivative **9a** (4-phenyl thiazole group) was more potent than thiazole derivative **9b** (4-bromophenyl thiazole group). This reflects that the incorporation of bromine in the structure of **9a** decreases its COX-2 inhibitory potential. This result is in concurrence with the earlier report [22]. However, the COX-2 inhibitory potential of the corresponding chrolo, fluoro, iodo, and nitro derivatives of **9b** should also be assessed for a better understanding of this observation. The thiazole derivative **8** (4-methyl-thiazole-5-carboxylate group) displayed a further decrease in the COX-2 inhibitory potential. This also indicates that the incorporation of methyl and carboxylate groups in the structure of **9a** decreases its COX-2 inhibitory activity.

In case of 4-thiazolidinone derivatives, the COX-2 inhibitory activity increases as **11** < **6a** < **18** < **6b** < **16a** < **10** < celecoxib < **17** < **12** < **16b**. The presence of the 5-dimethylaminomethylidene group (**11**), unsubstituted 4-thiazolidinone ring (**6a**), 5-methoxycarbonylmethylidene-3-phenyl group (**18**), methyl group (**6b**), 4-chlorophenylmethylidene group (**16a**), and 5-methoxycarbonylmethylidene group (**10**) at position 5 of the 4-thiazolidinone ring provides compounds with lesser or average COX-2 inhibitory action. However, when the chlorine of the **16a** is replaced with a methoxy group, a potent COX-2 inhibitor **16b** is obtained. This observation indicates that the presence of the electron donor group in these types of compounds increases the COX-2 inhibitory action. This reflection is also in concurrence with the earlier reports that the presence of the electron donor group may increase the COX-2 inhibitory action of thiazolidinone ring-bearing compounds [22,24]. The 2,3-disubstituted-4-thiazolidinone derivative (**17**) has been recognized as a potent inhibitor of COX-2. Some earlier reports also support this fact [26,28]. We also believe that this kind of other 2,3-disubstituted derivatives may provide potent COX-2 inhibitors. The isatin-bearing compound **12** also provided a potent inhibitor of COX-2. The incorporation of the isatin moiety is reported to potentiate the COX-2 inhibitory activity of a compound [33]. Recently, we reported the isomeric 4-thiazolidinone-bearing pyridazine derivatives, which had a methylidine linker joining the phenyl ring and the 4-thiazolidinone ring [30]. The presently reported 4-thiazolidinone-bearing pyridazine derivatives contain a methylidene hydrazinyl linker between the phenyl ring and the 4-thiazolidinone ring. A comparison of the COX-2 inhibitory activity among these isomeric compounds reveals that the incorporation of a methylidene hydrazinyl linker provides potent COX-2 inhibitors. This observation is in concurrence with our earlier report that states that compounds bearing a hydrazine moiety display higher COX-2 inhibition [30].

#### 2.2.2. In Vivo Anti-Inflammatory Activity

**Figure 2:**
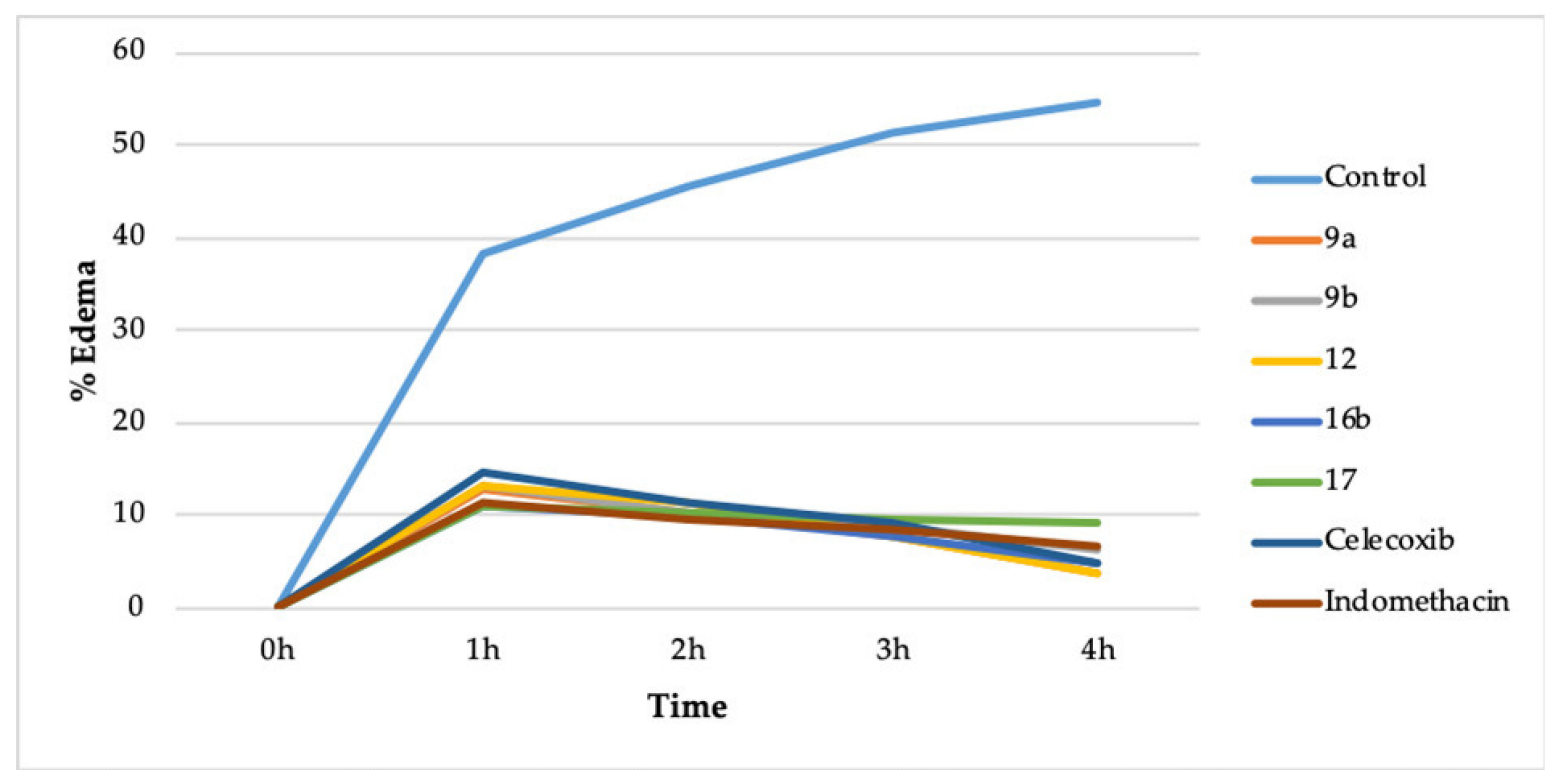
In vivo action of **9a**, **9b**, **12**, **16b**, **17**, celecoxib, and indomethacin.

The compounds **9a**, **9b**, **12**, **16b**, and **17** were chosen for the in vivo anti-inflammatory action because they displayed better COX-2 inhibition and lesser COX-1 inhibition. It was performed by the carrageenan-induced rat paw edema method [30,34]. A total of eight groups of rats were utilized, wherein each group comprised of six rats. The compounds were administered orally (10 mg/kg) (Table 2, Figure 2). 

The compounds **9a** (3.76%), **12** (3.70%), and **16b** (4.85%) reduced the % edema more than celecoxib (4.92%) and indomethacin (6.60%) after 4 h of drug administration. The compound **9b** (6.39%) reduced the % edema more than indomethacin (6.60%) but less than celecoxib (4.92%) after 4 h of drug administration. However, compound **17** (9.16%) reduced the % edema less than indomethacin (6.60%) and celecoxib (4.92%). The compounds **9a**, **9b**, **12**, **16b**, and **17** also have a comparable onset of action plus duration of action concerning celecoxib and indomethacin (Table 2, Figure 2).

Accordingly, it was determined that the novel pyridazine-based thiazole and 4-thiazolidinone derivatives are active lead compounds to develop future COX-2 inhibitors [22,24,26,28].

#### 2.2.3. Ulcerogenic Activity

**Figure 3:**
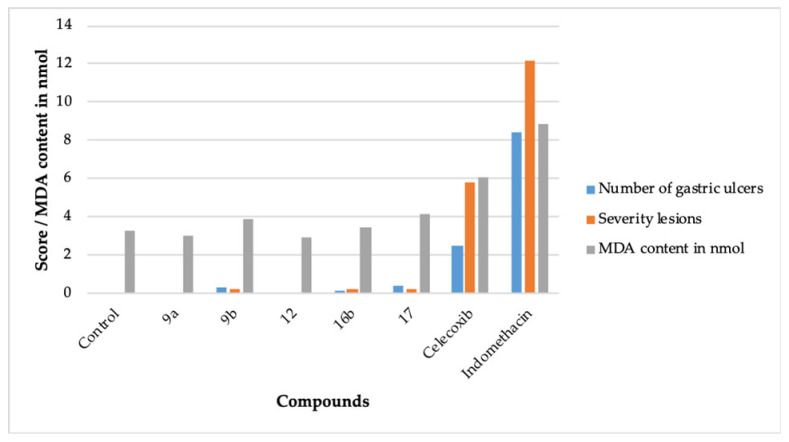
The ulcerogenic and lipid peroxidation activity effect of **9a**, **9b**, **11**, **15b**, **17**, celecoxib, and indomethacin.

The compromising gastric safety profile of the existing NSAIDs is a concern [6,7]. The ulcerogenic effects are more pronounced in non-specific COX inhibitors like indomethacin, whereas specific COX-2 inhibitors like celecoxib lack this side effect [6,10]. Accordingly, **9a**, **9b**, **12**, **16b**, and **17** were evaluated for their gastric safety profile. It was performed by the indomethacin-induced gastric erosion method [30]. A total of eight groups of rats were utilized, wherein each group comprised of six rats. The compounds were administered orally (10 mg/kg) (Table 3, Figure 3).

The compounds **9a** and **12** did not produce any ulcerogenic impact. The compounds **9b**, **16b**, and **17** also exhibited insignificant ulcerogenic effects. The negligible ulcerogenic effect produced by **9a**, **9b**, **12**, **16b**, and **17** is attributed to their higher COX-2 inhibitory potential plus their lesser potential to inhibit COX-1 (Table 1) [8,9]. It is well established that the presence of the -COOH group in NSAIDs aids in their ulcerogenic effect [6]. Another prospect of the insignificant ulcerogenic effect produced by **9a**, **9b**, **12**, **16b**, and **17** may be the absence of the -COOH moiety in their structure.

#### 2.2.4. Lipid Peroxidation Studies

The lipid peroxidation inhibitory activity of a compound makes it less ulcerogenic [35,36]. The lipid peroxidation in tissue increases the malondialdehyde (MDA) content in the tissue [35]. A decrease in the MDA content is a measure to assess the non-ulcerogenic effect of a compound. Accordingly, the compounds **9a**, **9b**, **12**, **16b**, and **17** were evaluated for their lipid peroxidation effects [35,36] (Table 3, Figure 3). The compounds **9a**, **9b**, **12**, **16b**, and **17** displayed a better lipid peroxidation profile than celecoxib and indomethacin, which provides concurrence to the ulcerogenic activity data (Table 3). 

### 2.3. Molecular Modelling

Molecular docking studies of **9a**, **9b**, **12**, **16b**, and **17** were carried out to validate the results of their biological activities [30,37,38,39] (Table 4).

The molecular docking data of celecoxib was described in our earlier publication [30]. The docking score (DS in kcal/mol) of compounds **9a** (DS = −10.8), **9b** (DS = −10.2), **12** (DS = −10.5), **16b** (DS = −10.6), and **17** (DS = −9.5) was superior to celecoxib (DS = −9.4) (Table 4). The superior docking scores of **9a**, **9b**, **12**, **16b**, and **17** support the in vitro COX-2 inhibitory analysis results of **9a** (IC_50_ = 15.50 nM, 114.77%), **9b** (IC_50_ = 17.50 nM, 101.65%), **12** (IC_50_ = 17.10 nM, 104.03%), **16b** (IC_50_ = 16.90 nM, 105.26%), and **17** (IC_50_ = 17.70 nM, 100.5%), and celecoxib (IC_50_ = 17.79 nM, 100%). Table 4 also mentions the hydrogen bonding pattern of celecoxib, **9a**, **9b**, **12**, **16b**, and **17** with the COX-2 protein. We also consider that the superior COX-2 inhibitory action of celecoxib, **9a**, **9b**, **12**, **16b**, and **17** is attributed to their hydrogen bonding interactions [30].

### 2.4. Physicochemical Parameters

The molecular properties of **9a**, **9b**, **12**, **16b**, and **17** were determined by the available online software, Molinspiration [40]. The calculated absorption (%ABS), number of H-bond acceptors (nON), number of H-bond donors (nOHNH), number of rotatable bonds (nrotb), and the topological polar surface area (tPSA) are provided in Table 5.

**Figure 4:**
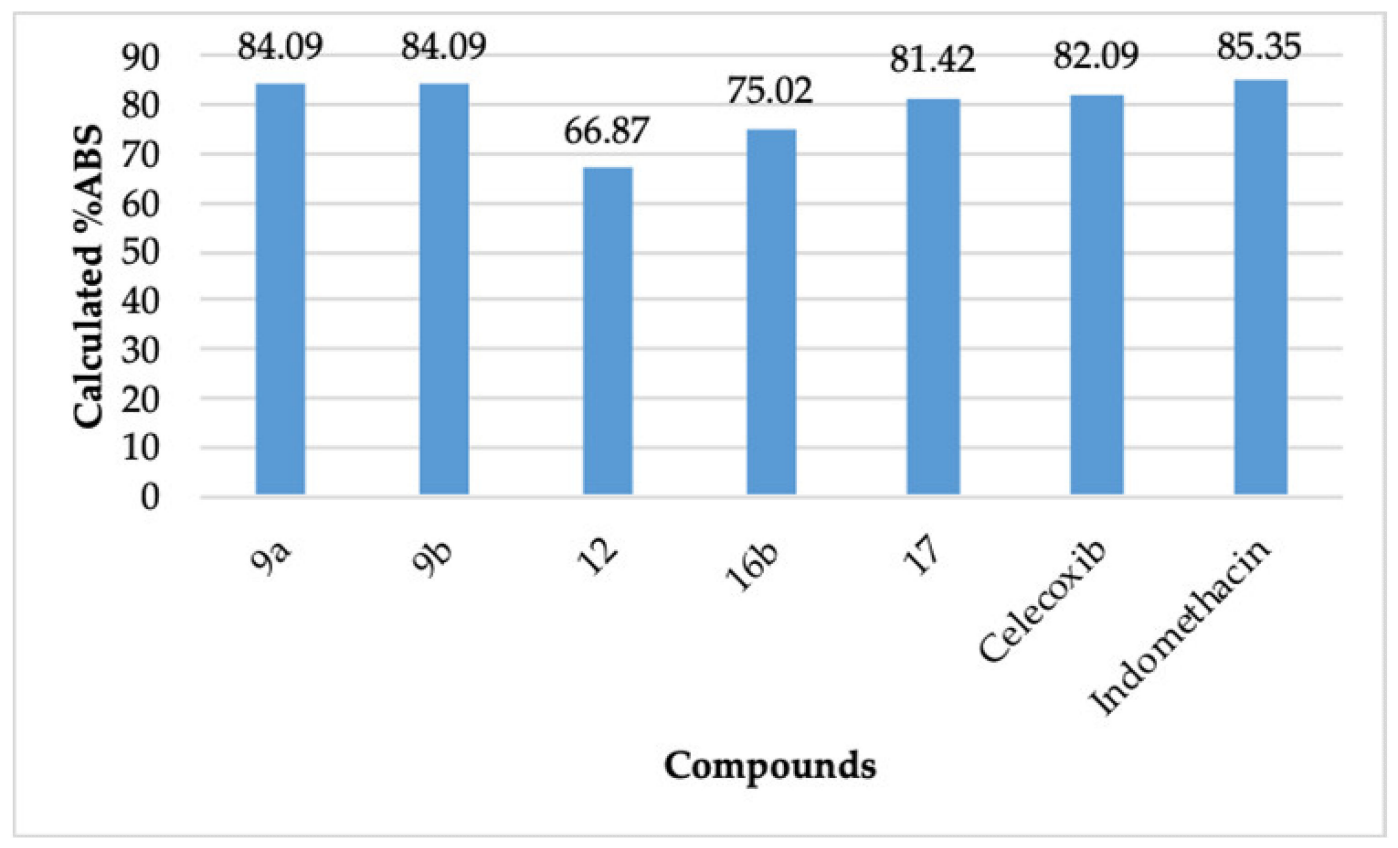
Calculated degree of absorption (%ABS) of **9a**, **9b**, **12**, **16b**, **17**, celecoxib, and indomethacin.

The compounds **9a**, **12**, **16b,** and **17** passed Lipinski’s rule [40,41]. This rule defines the molecular properties of a drug, which governs its pharmacokinetic performance, for example, the absorption of a drug. It also helps to evaluate a compound’s ability to be orally active based on its molecular properties [40,41], which are mentioned in Table 5. To pass Lipinski’s rule, a compound should not have more than one violation of the calculated properties. As per a recent report, Lipinski’s rule of five does not have any significant deficiency in defining the druggability of a compound and is still useful today [42]. It can be observed that compound **9a** displayed a better calculated absorption (%ABS = 84.09) than celecoxib (%ABS = 82.09) (Figure 4). The compound **9b** (%ABS = 84.09) also displayed a better calculated absorption than celecoxib. The compound **9b** did not pass Lipinski’s rule. However, there are many clinically used natural compounds and drugs that violate Lipinski’s rule [42]. The compound **12** (%ABS = 66.87), compound **16b** (%ABS = 75.02), and compound **17** (%ABS = 81.42) also exhibited appreciable calculated absorption. These observations revealed that the compounds **9a**, **9b, 12**, **16b,** and **17** possess not only better COX-2 inhibitory action than indomethacin and celecoxib but also possess a promising pharmacokinetic/physicochemical profile.

## 3. Materials and Methods

The uncorrected melting points, FTIR spectra (KBr), NMR spectra (^1^H-NMR & ^13^C-NMR), mass spectra, and elemental analyses were obtained by Gallenkamp apparatus (MFB-595- 010M; Weiss Gallenkamp, London, UK), Shimadzu 440 spectrometer (Shimadzu, Tokyo, Japan), Varian Gemini 500 MHz spectrometer (Palo Alto, CA, USA), GCMS/QP 1000 Ex mass spectrometer (70 eV) (Shimadzu, Tokyo, Japan), and the VARIO El Elementer apparatus (Langenselbold, Germany), respectively. The melting points are mentioned in °C, IR signals are mentioned as (KBr, *ν* in cm^−1^), ^1^H-NMR signals are mentioned as (500 MHz, DMSO-*d*_6_; *δ* in ppm), ^13^C-NMR signals are mentioned as (125 MHz, DMSO-*d*_6_, *δ* in ppm), and the mass peaks (MS) are represented as (*m*/*z*). The intermediates **2**, **3**, **4b**, and **4c** were reported in our previous publications [30,43]. SciFinder was used to ascertain the novelty of **4a**, **6a, 6b**, **8**, **9a, 9b**, **10**, **11**, **12**, **16a**, **16b, 17**, and **18**. The preparation methods of **4a**, **6a**, **6b**, **8**, **9a**, **9b**, **10**, **11**,**12**, **16a**, **16b**, **17**, and **18** are depicted in Scheme 1, Scheme 2, Scheme 3, Scheme 4 and Scheme 5.

### 3.1. Chemistry

General preparation of **4a**–**c**

A mixture comprising **3** (3.62 mmoles), hydrazine hydrate or thiosemicarbazide or phenylthiosemicarbazide (3.62 mmoles), AcOH (5 mL), and EtOH (50 mL) was refluxed at 80 °C for 3–5 h. The separated precipitate was filtered and recrystallized by MeOH. The compounds **4b** and **4c** were reported in our previous publications [30].

*2-(4-(Hydrazineylidenemethyl)phenyl)-6-phenylpyridazin-3(2H)-one* (**4a**)*:* Yield (75%); M.P.: 235–236 °C; IR: 3371 & 3216 (NH_2_), 3063, 1662 (C=O); ^1^H-NMR: 6.98 (s, 2H, NH_2_), 7.14 (d, 1H, Ar-H), 7.42 (q, 3H, Ar-H), 7.61 (dd, 4H, Ar-H), 7.79 (s, 1H, azomethine-H), 7.88 (d, 2H, Ar-H), 8.05 (d, 1H, Ar-H); ^13^C-NMR: 159.06 (C=O), 144.59, 140.95, 137.56, 136.59, 134.64, 131.45, 131.27, 129.99, 129.37 (2C), 128.45 (2C), 126.46 (2C), 126.08 (2C), 125.52 (2C); Mass: 290 (M^+^, 100% base peak); Anal. Calcd. for C_17_H_14_N_4_O: C, 70.33; H, 4.86; N, 19.30. Found: C, 70.22; H, 4.70; N, 19.24.

General synthesis of **6a**, **6b**, **7**, **9a**, and **9b**

A mixture comprising **4b** (10 mmoles), ethyl 2-chloroacetate (10 mmoles) or ethyl 2-chloropropanoate (10 mmoles) or ethyl 2-chloro-3-oxobutanoate (10 mmoles) or 2-bromo-1-phenylethan-1-one (10 mmoles) or 2-bromo-1-(4-bromophenyl)ethan-1-one (10 mmoles) and CH_3_COONa (20 mmoles) in 40 mL of AcOH was heated to 125 °C for 4–6 h. The precipitate was collected and recrystallized by dioxane.

*2-(2-(4-(6-Oxo-3-phenylpyridazin-1(6H)-yl)benzylidene)hydrazineyl)thiazol-4(5H)-one* (**6a**)*:* Yield (65%); M.P.: 250–252 °C; IR: 3150 (NH), 2942, 2850, 1720 & 1665 (C=O), 1584; ^1^H-NMR: 3.92 (s, 2H, -S-CH_2_-), 7.20 (d, 2H, Ar-H), 7.48 (q, 3H, Ar-H), 7.80 (d, 2H, Ar-H), 7.89 (d, 2H, Ar-H), 8.14 (d, 2H, Ar-H), 8.48 (s, 1H, azomethine-H), 12.12 (s, 1H, NH); ^13^C-NMR: 170.43 (C=O), 159.06 (C=O), 155.87, 154.58, 144.88, 143.59, 134.57, 134.15, 131.64, 130.14 (2C), 129.43 (2C), 128.25 (2C), 126.56 (2C), 126.34 (2C), 33.54 (S-CH_2_-); Mass: 389 (M^+^), 274 (100% base peak); Anal. Calcd. for C_20_H_15_N_5_O_2_S: C, 61.68; H, 3.88; N, 17.98. Found: C, 61.47; H, 3.76; N, 17.89.

*5-Methyl-2-(2-(4-(6-oxo-3-phenylpyridazin-1(6H)-yl)benzylidene)hydrazineyl)thiazol-4(5H)-one* (**6b**)*:* Yield (67%): M.P.: 285–286 °C; IR: 3115 (NH), 3059, 1714 & 1668 (C=O), 1595; ^1^H-NMR: 1.57 (d, 3H, CH_3_), 4.22 (q, 1H, CH), 7.22 (d, 1H, Ar-H), 7.46 (q, 3H, Ar-H), 7.80 (d, 2H, Ar-H), 7.94 (dd, 4H, Ar-H), 8.14 (d, 1H, Ar-H), 8.48 (s, 1H, azomethine-H), 12.02 (s, 1H, NH); ^13^C-NMR: 174.21 (C=O), 159.06 (C=O), 155.91, 153.47, 144.89, 143.61, 134.58, 134.15, 131.65, 130.14 (2C), 129.43 (2C), 128.26 (2C), 126.57 (2C), 126.35 (2C), 42.71 (-S-CH_2_-), 19.20 (-CH_3_); Mass: 403 (M^+^), 274 (100% base peak); Anal. Calcd. for C_21_H_17_N_5_O_2_S: C, 62.52; H, 4.25; N, 17.36. Found: C, 62.50; H, 4.22; N, 17.35.

*Ethyl 4-methyl-2-(2-(4-(6-oxo-3-phenylpyridazin-1(6H)-yl)benzylidene)hydrazineyl) thiazole-5-carboxylate* (**8**)*:* Yield (74%): M.P.: 245–246 °C; IR: 3115 (NH), 3059, 1715 & 1665 (C=O), 1595; ^1^H-NMR: 1.43 (s, 3H, CH_3_), 2.26 (s, 3H, CH_3_), 4.20 (q, 2H, CH_2_), 7.24 (d, 2H, Ar-H), 7.49–7.60 (m, 4H, Ar-H), 8.05 (m, 4H, Ar-H), 8.20 (d, 1H, Ar-H), 8.49 (s, 1H, azomethine-H), 9.77 (s, 1H, NH); ^13^C-NMR: 170.51 (C=O), 167.15 (C=O), 160.95, 160.02, 159.13, 157.72, 145.10, 143.47, 135.19, 134.58, 134.45, 133.39, 131.78, 131.14, 130.98, 130.19, 129.47, 128.93, 126.65, 126.19, 116.91, 60.89 (-O-CH_2_-), 17.27 (-CH_3_), 14.75 (Ester -CH_3_); Mass: 459 (M^+^), 274 (100% base peak); Anal. Calcd. for C_24_H_21_N_5_O_3_S: C, 62.73; H, 4.61; N, 15.24. Found: C, 62.70; H, 4.55; N, 15.24.

*6-Phenyl-2-(4-((2-(4-phenylthiazol-2-yl)hydrazinylidene)methyl)phenyl)pyridazin-3(2H)-one* (**9a**)*:* Yield (72%): M.P.: 280–281 °C; IR: 3115 (NH), 3059, 1666 (C=O), 1595; ^1^H-NMR: 7.04–7.50 (m, 9H, Ar-H + thiazole-H), 7.79–8.11 (m, 9H, Ar-H + azomethine-H), 12.28 (s, 1H, NH); ^13^C-NMR: 168.60 (C=O), 159.07, 144.82, 142.46, 140.67, 135.13, 134.64, 134.44, 132.37, 131.60, 131.52, 130.10, 129.43, 129.08 (2C), 128.02 (2C), 126.79 (2C), 126.55 (2C), 126.34 (2C), 126.0 (2C), 104.33; Mass: 449 (M^+^), 77 (100% base peak); Anal. Calcd. for C_26_H_19_N_5_OS: C, 69.47; H, 4.26; N, 15.58. Found: C, 69.45; H, 4.25; N, 15.55.

*2-(4-((2-(4-(4-Bromophenyl)thiazol-2-yl)hydrazineylidene)methyl)phenyl)-6-phenylpyridazin-3(2H)-one* (**9b**)*:* Yield (70%): M.P.: 261–262 °C; IR: 3115 (NH), 3059, 1670 (C=O), 1595; ^1^H-NMR: 7.20–8.13 (m, 17H, Ar-H + thiazole-H5 + azomethine-H), 12.31 (s, 1H, NH); ^13^C-NMR: 168.78 (C=O), 159.08, 149.92, 144.83, 142.53, 141.64, 140.87, 134.66, 134.36, 132.02, 131.62, 131.51, 130.11, 129.44, 128.59 (2C), 128.04, 127.91 (2C), 126.84, 126.57 (2C), 126.34, 126.15, 121.03, 105.24; Mass: 527 (M^+^), 529 (M^+^ + 2), 274 (100% base peak); Anal. Calcd. for C_26_H_18_BrN_5_OS: C, 59.10; H, 3.43; N, 13.25. Found: C, 59.11; H, 3.42; N, 13.21.

*Synthesis of Methyl2-(4-oxo-2-((4-(6-oxo-3-phenylpyridazin-1(6H)-yl)benzylidene)hydrazineylidene) -thiazolidin-5-ylidene)acetate* (**10**). A mixture comprising **4b** (5 mmoles), dimethyl acetylene dicarboxylate (5 mmol), and MeOH (30 mL) was reacted at 80 °C for 1 h. The resultant residue was cooled at 25 °C. The solid was filtered, and recrystallized by dioxane. Yield (75%): M.P.: 290–291 °C; IR: 3183 (NH), 2963, 2776, 1724 (C=O), 1710 & 1678 (C=O), 1614; ^1^H-NMR: 3.77 (s, 3H, OMe), 6.69 (s, 1H, CH=C), 7.24–7.39 (m, 8H, Ar-H), 7.75–7.92 (m, 3H, Ar-H), 8.34 (s, 1H, azomethine-H), 12.29 (hump, 1H, NH); ^13^C-NMR: 175.93 (C=O), 167.57 (C=O), 161.05 (C=O), 159.43, 159.13, 157.51, 155.24, 145.11, 143.50, 134.58, 134.32, 132.42, 132.30, 131.78, 130.19, 129.48, 129.04, 128.71, 126.66, 126.12, 122.12, 121.93, 56.37 (-OCH_3_); Mass: 459 (M^+^), 274 (100% base peak); Anal. Calcd. for C_23_H_17_N_5_O_4_S: C, 60.12; H, 3.73; N, 15.24. Found: C, 60.10; H, 3.69; N, 15.22.

*Synthesis of 5-((dimethylamino)methylene)-2-(2-(4-(6-oxo-3-phenylpyridazin-1(6H)-yl) benzylidene) hydrazineyl) thiazol-4(5H)-one* (**11**). A mixture comprising **6a** (0.01 mole), dimethylformamide-dimethylacetal (DMF-DMA) (0.01 moles) and dioxane (30 mL) was refluxed for 4 h. The resultant residue was cooled at 25 °C. The solid was isolated, and recrystallized with EtOH-dioxane mixture (1:1). Yield (70%): M.P.: 284–285 °C; IR: 3183 (NH), 2963, 2776, 1724 & 1678 (C=O), 1614; ^1^H-NMR: 2.94 & 3.13 (2s, 6H, N(CH_3_)_2_), 7.18 (d, 1H, Ar-H), 7.49 (brs, 3H, Ar-H + methine-H), 7.74 (d, 3H, Ar-H), 7.94 (q, 3H, Ar-H), 8.12 (d, 2H, Ar-H), 8.25 (s, 1H, azomethine-H), 12.34 (s, 1H, NH); ^13^C-NMR: 172.72 (C=O), 167.35 (C=O), 159.07, 157.10, 144.91, 144.85, 144.59, 143.74, 143.33, 134.57, 134.47, 134.04, 131.63, 130.14, 129.43, 128.38, 128.06, 127.69, 126.55, 126.35, 126.27, 42.37 (2C, 2CH_3_); Mass: 444 (M^+^), 274 (100% base peak); Anal. Calcd. for C_23_H_20_N_6_O_2_S: C, 62.15; H, 4.54; N, 18.91. Found: C, 62.13; H, 4.52; N, 18.91.

*Synthesis of 2-(2-(4-(6-oxo-3-phenylpyridazin-1(6H)-yl)benzylidene)hydrazineyl)-5- (2-oxoindolin-3-ylidene) thiazol-4(5H)-one* (**12**). A mixture comprising **6a** (0.01 mole), indoline-2,3-dione (0.01 mole), piperidine (0.5 mL), and dioxane (40 mL) was refluxed for 2 h. The solid was filtered hot, and recrystallized with dioxane. Yield (77%); M.P.: 255–256 °C; IR: 3169 & 3149 (NH), 3054, 2973, 1723 (C=O), 1705 & 1672 (C=O), 1613; ^1^H-NMR: 6.76 (d, 1H, Ar-H), 6.93 (d, 1H, Ar-H), 7.05 (d, 2H, Ar-H), 7.22 (d, 2H, Ar-H), 7.50 (brs, 3H, Ar-H), 7.87, 7.92 (2brs, 5H, Ar-H), 8.18 (d, 1H, Ar-H), 8.36 (s, 1H, azomethine-H), 11.20, 12.51 (2s, 2H, 2NH); ^13^C-NMR: 175.43 (C=O), 168.22 (C=O), 161.15 (C=O), 159.53, 159.23, 157.51, 155.34, 145.12, 143.60, 134.58, 134.38, 132.33, 132.24, 131.68, 130.29, 129.48 (2C), 129.14 (2C), 128.71 (2C), 126.67 (3C), 126.22 (2C), 122.14, 121.92; Mass: 518 (M^+^), 274 (100% base peak); Anal. Calcd. for C_28_H_18_N_6_O_3_S: C, 64.86; H, 3.50; N, 16.21. Found: C, 64.70; H, 3.37; N, 16.10. 

General Synthesis of **16a** and **16b**

A mixture comprising **6a** (10 mmoles), the requisite cinnamonitrile (10 mmoles), piperidine (0.5 mL), and dioxane (40 mL) was refluxed for 4–6 h. The solid produced was collected and recrystallized using dioxane. Alternatively, **16a** and **16b** can also be prepared by reacting equimolar quantities of **6a** with appropriate benzaldehyde.

*5-(4-Chlorobenzylidene)-2-(2-(4-(6-oxo-3-phenylpyridazin-1(6H)-yl)benzylidene)-hydra-zinyl)thiazol-4(5H)-one* (**16a**)*:* Yield (81%): M.P.: 297–298 °C; IR: 3160 (NH), 2940, 1729 & 1670 (C=O), 1619; ^1^H-NMR: 7.24 (d, 1H, Ar-H), 7.51 (brs, 3H, Ar-H), 7.84 (d, 2H, Ar-H), 7.97 (brs, 6H, Ar-H + benzylidene-H), 8.14, 8.24 (2d, 4H, Ar-H), 8.40 (s, 1H, azomethine-H), 10.79 (s, 1H, NH); ^13^C-NMR: 180.80 (C=O), 176.51 (C=O), 174.15, 159.08, 156.90, 149.16, 144.77, 142.32, 140.81, 136.49, 135.88 (2C), 134.93 (2C), 134.67 (2C), 129.43 (4C), 126.55 (3C), 126.53 (2C), 120.69, 120.30; Mass: 511 (M^+^), 510 (100% base peak); Anal. Calcd. for C_27_H_18_ClN_5_O_2_S: C, 63.34; H, 3.54; N, 13.68. Found: C, 63.25; H, 3.48; N, 13.54.

*5-(4-Methoxybenzylidene)-2-(2-(4-(6-oxo-3-phenylpyridazin-1(6H)-yl)benzylidene)hydra-zineyl)thiazol-4(5H)-one* (**16b**)*:* Yield (83%): M.P.: >300 °C; IR: 3164 (NH), 1700 & 1665 (C=O), 1613; ^1^H-NMR: 3.91 (s, 3H, OCH_3_), 6.94 (d, 2H, Ar-H), 7.24 (d, 2H, Ar-H), 7.50–7.58 (2brs, 5H, Ar-H), 7.80–8.12 (3brs, 6H, Ar-H + CH-benzylidene), 8.18 (d, 1H, Ar-H), 8.50 (s, 1H, azomethine-H), 11.12 (s, 1H, NH); ^13^C-NMR: 168.27 (C=O), 166.11 (C=O), 165.48, 159.09, 155.46, 155.17, 144.76, 134.65, 131.61 (2C), 130.98 (2C), 130.11 (2C), 129.44 (3C), 129.16 (3C), 127.21 (2C), 126.55 (3C), 126.16 (2C), 55.68 (-OCH_3_); Mass: 507 (M^+^), 506 (100% base peak); Anal. Calcd. for C_28_H_21_N_5_O_3_S: C, 66.26; H, 4.17; N, 13.80. Found: C, 66.15; H, 4.15; N, 13.74.

*Synthesis of 2-((4-(6-oxo-3-phenylpyridazin-1(6H)-yl)benzylidene)hydrazineylidene)-3-phenyl thiazolidin-4-one* (**17**). A mixture comprising **4c** (10 mmoles), ethyl 2-chloroacetate (10 mmoles), CH_3_COONa (20 mmoles), and AcOH (30 mL) was heated at 125 °C for 4 h. The produced solid was isolated and recrystallized by dioxane. Yield (72%): M.P.: 283–284 °C; IR: 3172 (NH), 2953, 2756, 1730 & 1699 (C=O), 1621; ^1^H-NMR: 4.13 (s, 2H, CH_2_), 7.19 (d, 2H, Ar-H), 7.41-7.54 (m, 7H, Ar-H), 7.78 (d, 1H, Ar-H), 7.87 (d, 2H, Ar-H), 7.93 (d, 2H, Ar-H), 8.13 (d, 2H, Ar-H), 8.40 (s, 1H, azomethine-H); ^13^C-NMR: 172.53 (C=O), 165.0 (C=O), 159.06, 157.36, 144.88, 135.51, 134.55, 131.63, 130.16, 129.59 (2C), 129.43 (2C), 129.19 (2C), 128.76 (2C), 128.59 (2C), 128.37 (2C), 126.56 (2C), 126.39 (2C), 32.85 (-S-CH_2_-); Mass: 465 (M^+^), 274 (100% base peak); Anal. Calcd. for C_26_H_19_N_5_O_2_S: C, 67.08; H, 4.11; N, 15.04. Found: C, 67.0; H, 4.03; N, 14.98.

*Synthesis of Methyl2-(4-oxo-2-((4-(6-oxo-3-phenylpyridazin-1(6H)-yl)benzylidene)hydrazinylidene) -3-phenylthiazolidin-5-ylidene)acetate* (**18**). A mixture comprising **4c** (5 mmoles), dimethyl acetylenedicarboxylate (5 mmol), and MeOH (30 mL) was heated to 80 °C for 1 h. The resultant mixture was cooled to 25 °C. The solid separated was collected and recrystallized by ethanol-dimethylformamide (DMF) mixture (1:1). Yield (77%): M.P.: 277–278 °C; IR: 2953, 2756, 1730 (C=O), 1699 & 1670 (C=O), 1621; ^1^H-NMR: 3.84 (s, 3H, OMe), 6.84 (s, 1H, methine-H), 7.21 (d, 1H, Ar-H), 7.47–7.59 (m, 8H, Ar-H), 7.82 (d, 2H, Ar-H), 7.92 (d, 4H, Ar-H), 8.18 (d, 1H, Ar-H), 8.55 (s, 1H, azomethine-H); ^13^C-NMR: 169.90 (C=O), 166.48 (C=O), 159.42 (C=O), 157.13, 156.46, 144.94, 142.13, 134.54, 133.44, 131.67, 130.18 (2C), 129.64 (3C), 129.44 (3C), 128.80 (3C), 128.70 (2C), 126.58 (3C), 126.50 (2C), 53.08 (-OCH_3_); Mass: 535 (M^+^), 274 (100% base peak); Anal. Calcd. for C_29_H_21_N_5_O_4_S: C, 65.04; H, 3.95; N, 13.08. Found: C, 65.0; H, 3.91; N, 13.02.

### 3.2. Biological Activity

#### 3.2.1. In Vitro COX-1 and COX-2 Inhibition Assay

The compounds **4a**, **6a**, **6b**, **8**, **9a**, **9b**, **10**, **11**, **12**, **16a**, **16b**, **17**, and **18** were exposed to their COX-1/COX-2 inhibitory action by a 10-fold dilution strategy (1–10^−4^ μg/mL) utilizing dimethylsulfoxide (DMSO) [30]. The test packs of the human COX-1/COX-2 were obtained from Cayman Chemicals (560131, Ann Arbor, MI, USA). The supplier’s instructions were followed to prepare the reagents as well as to perform the experiment. Briefly, samples (20 μL), the COX-1 and COX-2 enzyme (10 μL), and heme (10 μL) were mixed with the buffer solution (160 μL), which was supplied with the kits. The resultant combination was incubated at 37 °C in a water bath for 10 min, and arachidonic acid (10 μL) was added to initiate the COX reaction. A saturated solution of stannous chloride (30 μL) was added after 2 min to halt the COX reaction. The resultant mixture was incubated at ambient temperature for 5 min. The Prostaglandin-2α (PGF2α) developed after the COX reaction was measured through ELISA. The samples were shifted to a 96-well plate and incubated for 18 h at 25 °C. The plate was washed to get rid of the unbound components. The Ellman’s reagent (200 μL), containing the acetylcholine substrate, was mixed and further incubated at 25 °C for 1–1.5 h till the absorbance (410 nm) of the *B_o_* well was in the range of 0.3 to 0.8 A.U. The plate was read through the ELISA reader. The IC_50_ values of COX-1 and COX-2 were determined by the regression analysis.

#### 3.2.2. In Vivo Anti-Inflammatory Activity

The compounds **9a**, **9b**, **12**, **16b**, and **17** were evaluated for anti-inflammatory action utilizing Wistar rats (130–150 g) by following the rat paw edema method [30,34]. The animal approval (IAEC/KSOP/E/18/12) was obtained from the CPCSEA. A total of eight groups of rats were utilized, wherein each group comprised of 6 rats. The compounds (test group), celecoxib (standard group), and indomethacin (standard group) were administered orally (10 mg/kg) as a 10% Tween-80 solution. Saline solution (1 mL) was given to the control group. The carrageenan solution (1%, 0.1 mL) was administered by injection after 1 h in the right hind paw of the rats in the test, standard, and the control groups. The volume of the paws was calculated after the carrageenan injection at 0, 1, 2, 3, and 4 h by a plethysmometer. The % edema was calculated as follows:

$$\%\;edema=\frac{paw\;diameter\;after\;carrageenan-paw\;diameter\;before\;carrageenan}{paw\;diameter\;before\;carrageenan}\times 100.\;$$

#### 3.2.3. Gastric Ulcerogenic Activity

The eight groups of rats were fasted for 18 h, wherein each group consisted of 6 rats. The compounds (**9a**, **9b**, **12**, **16b**, and **17**), celecoxib, and indomethacin were given orally (10 mg/kg) as 10% Tween-80 solution. Saline solution (1 mL) was given to the control group. The animals were sacrificed after 4 h to isolate their stomachs. A longitudinal cut was made along the greater curvature, and the presence of ulcers was evaluated. The counting of ulcers was marked as 0 (no ulcer) to 5 (≥ 3 ulcers) [30].

#### 3.2.4. Lipid Peroxidation Inhibitory Activity

It was carried out on compounds **9a**, **9b**, **12**, **16b**, **17,** celecoxib, and indomethacin after the ulcerogenic activity [35,36]. The gastric mucosa (100 mg) was scraped with two glass slides and homogenized in ice-cold potassium chloride (1.8 mL of 1.15% KCl). Sodium dodecyl sulphate (0.2 mL of 8.1% SDS), acetate buffer (pH = 3.5, 1.5 mL), and thiobarbituric acid (1.5 mL of 0.8% TBA) was mixed with the homogenate, and the mixture was heated to 95 °C for 1 h. The obtained mixture was extracted with a 5-mL mixture (15:1 v/v) of n-butanol and pyridine and centrifuged at 4000 rpm for 10 min. The supernatant liquid was separated, and its absorbance was measured at 532 nm using a spectrophotometer. A standard linear curve (absorbance vs. concentration in nM) was prepared using MDA tetrabutylammonium salt. The lipid peroxidation was calculated from the standard curve. The results are expressed as nmol MDA 100 mg^−1^ tissues.

### 3.3. Molecular Docking

The molecular docking of **9a**, **9b**, **12**, **16b**, **17,** and celecoxib was performed by the method reported in our earlier report [30]. This study was conducted on the HP^®^ computer system (Intel^®^ core i5-4570 CPU, 3.20 GHz, Window 10). ChemDraw was used to design the compounds, wherein ChemBio3D Ultra was used to generate 3-D conformations. The 3-D structure of COX-2 protein (PDB entry 5KIR) was retrieved from PDB [37,38,39]. The Auto Dock and the Auto Dock Vina were utilized to generate the data. The detailed procedure was demonstrated in our earlier report [30].

### 3.4. Physicochemical Parameters

The concept of “Lipinski’s rule” [40,41] was applied to **9a**, **9b**, **12**, **16b**, and **17**. The computational molecular properties like the molecular weight, number of H-bond acceptors and donors, log P, number of rotatable-bonds, and molecular polar surface area were determined by the available online software, Molinspiration [40,41]. The degree of absorption (%ABS) was computed as follows [44,45]:

%ABS = 109 − (0.345 × tPSA)

### 3.5. Statistical Analysis

It was performed by the SPSS software. The *p* values, N values, mean values, and standard deviation values are mentioned at the desirable places of the manuscript.

## 4. Conclusions

Five compounds, **9a**, **9b**, **12**, **16b**, and **17**, demonstrated superior COX-2 inhibition than celecoxib. These compounds had a similar onset/duration of action to celecoxib. The compounds **9a** and **12** were devoid of any ulcerogenic effect, whereas **9b**, **16b,** and **17** showed insignificant ulcerogenic effects. The compounds **9a**, **9b**, **12**, **16b**, and **17** also displayed a better lipid peroxidation profile than indomethacin and celecoxib. They also demonstrated a considerable calculated absorption. The compounds **9a**, **9b, 11**, **16b,** and **17** are thus recognized and postulated as non-ulcerogenic COX-2 inhibitors with promising physicochemical parameters and gastric safety profile. These compounds may be useful candidates to combat diseases caused by higher levels of COX-2 like gout, ankylosing spondylitis, osteoarthritis, rheumatoid arthritis, Alzheimer’s disease, ulcerative colitis, depression, epilepsy, irritable bowel diseases, kidney injury, cancer, asthma, hepatitis, pancreatitis, and atherosclerosis.

## Figures and Tables

**Table 1:** In vitro cyclooxygenase inhibitory effect (N = 3, Mean ± SD) of **4a**, **6a**, **6b**, **8**, **9a**, **9b**, **10**, **11**, **12**, **16a**, **16b**, **17**, and **18**.

Compound	COX-1 (IC_50_, nM *)	%COX-1 Inhibition	COX-2 (IC_50_, nM *)	%COX-2 Inhibition	SI	%SI
**4a**	235 ± 0.11	93.61	26.19 ± 0.50	67.92	8.97	49.88
**6a**	260 ± 0.22	84.61	25.10 ± 0.15	70.87	10.35	57.56
**6b**	280 ± 0.50	78.57	22.75 ± 0.10	78.19	12.30	68.40
**8**	250 ± 0.12	88.0	24.18 ± 0.14	73.57	10.33	57.45
**9a**	330 ± 0.28	66.66	15.50 ± 0.55	114.77	21.29	118.40
**9b**	275 ± 0.42	80.0	17.50 ± 0.60	101.65	15.71	87.37
**10**	285 ± 0.15	77.19	22.11 ± 0.55	80.46	12.89	71.69
**11**	270 ± 0.24	81.48	25.11 ± 0.11	70.84	10.75	59.78
**12**	295 ± 0.28	74.57	17.10 ± 0.19	104.03	17.25	95.93
**16a**	275 ± 0.10	80.0	22.16 ± 0.44	80.27	12.40	68.96
**16b**	315 ± 0.33	69.84	16.90 ± 0.16	105.26	18.63	103.61
**17**	285 ± 0.16	77.19	17.70 ± 0.18	100.5	16.10	89.54
**18**	255 ± 0.20	86.27	23.15 ± 0.50	76.84	11.01	61.23
**Celecoxib**	320 ± 0.01	68.75	17.79 ± 0.69	100.0	17.98	100.0
**Indomethacin**	220 ± 0.01	100.0	67.72 ± 0.62	26.26	3.24	18.02

* *p <* 0.5 (SPSS); SI (Selectivity index): IC_50_ for COX-1/IC_50_ for COX-2.

**Table 2:** In vivo anti-inflammatory activity of **9a**, **9b**, **12**, **16b**, and **17**.

Compound	0 h	1 h		2 h		3 h		4 h	
	PD *	PD	% Edema	PD	% Edema	PD	% Edema	PD	% Edema
**Control**	3.40 ± 0.20	4.70 ± 0.42	38.23	4.95 ± 0.21	45.58	5.15 ± 0.10	51.47	5.25 ± 0.32	54.41
**9a**	3.45 ± 0.20	3.89 ± 0.14	12.75	3.81 ± 0.12	10.43	3.72 ± 0.14	7.82	3.58 ± 0.36	3.76
**9b**	3.44 ± 0.18	3.89 ± 0.10	13.08	3.80 ± 0.28	10.46	3.74 ± 0.16	8.72	3.66 ± 0.40	6.39
**12**	3.51 ± 0.16	3.98 ± 0.62	13.39	3.91 ± 0.10	11.39	3.78 ± 0.22	7.69	3.64 ± 0.15	3.70
**16b**	3.50 ± 0.32	3.89 ± 0.21	11.14	3.85 ± 0.30	10.0	3.77 ± 0.50	7.71	3.67 ± 0.42	4.85
**17**	3.49 ± 0.13	3.88 ± 0.10	11.17	3.85 ± 0.13	10.31	3.82 ± 0.12	9.45	3.81 ± 0.14	9.16
**Celecoxib**	3.45 ± 0.22	3.95 ± 0.22	14.49	3.84 ± 0.22	11.30	3.77 ± 0.33	9.27	3.62 ± 0.14	4.92
**Indomethacin**	3.48 ± 0.44	3.88 ± 0.26	11.49	3.81 ± 0.40	9.48	3.77 ± 0.14	8.33	3.71 ± 0.33	6.60

* *p* < 0.5 (SPSS); PD: Paw diameter (mm).

**Table 3:** The gastric safety profile of **9a**, **9b**, **12**, **16b**, and **17**.

Group	Score		MDA Content in nmol (100 mg Tissue)
	No of Gastric Ulcers *	Severity Lesions *	
**Control**	0	0	3.24 ± 0.32
**9a**	0	0	3.05 ± 0.12
**9b**	0.33 ± 0.38	0.26 ± 0.16	3.89 ± 0.50
**12**	0	0	2.95 ± 0.44
**16b**	0.12 ± 0.30	0.18 ± 0.22	3.45 ± 0.28
**17**	0.42 ± 0.10	0.20 ± 0.50	4.11 ± 0.18
**Celecoxib**	2.51 ± 0.32	5.82 ± 0.44	6.11 ± 0.50
**Indomethacin**	8.40 ± 0.60	12.15 ± 0.10	8.90 ± 0.18

* *p* < 0.5, Mean ± SE, (SPSS).

**Table 4:** Molecular docking data of **9a**, **9b**, **12**, **16b**, and **17**.

Compound	Binding Affinity (kcal/mol)	Hydrogen Bonding Interacting Residues *	Other Interacting Residues
**Celecoxib**	−9.4	MET 522 (-NH of sulphonamide group), ARG 120 (Fluorine of -CF_3_ group)	TRP 387, LEU 352, ALA 527, PHE 518, VAL 349, VAL 523, LEU 359
**9a**	−10.8	CYS 47 (imine group of -CH=N-), TYR 30 (C=O of the pyridazinone)	TYR 136, GLY 135, PRO 156, PRO 154, PRO 153, LYS 468
**9b**	−10.2	CYS 47 (imine group of -CH=N-), TYR 30 and PRO 153 (C=O of the pyridazinone)	PRO 514, PRO 156, SER 353, LYS 468
**12**	−10.5	ARG 44 (2^o^-amino group of =N-NH- moiety), HIS 122 (2^o^-amino group of isatin), TYR 130 (phenyl ring)	ASP 125, LYS 137, VAL 46, PRO 153, CYS 47, CYS 36
**16b**	−10.6	TYR 130 (C=O of the pyridazinone), ASN 34 (C=O of the thiazolidinoe)	TYR 136, ARG 469, LEU 152, PRO 153, CYS 47, CYS 36
**17**	−9.5	LYS 473 (phenyl ring)	VAL 89, PRO 84, TYR 115, GLU 524, PRO 86

* Dark green bonds in the Supplementary figures of the molecular docking.

**Table 5:** The physicochemical parameters of **9a**, **9b**, **12**, **16b**, **17**, celecoxib, and indomethacin.

Compound	%ABS	tPSA	nrotb ≤ 10	nON ≤ 10	nOHNH ≤ 5	miLogP ≤ 5	MW ≤ 500	n Violations ≤ 1
**9a**	84.09	72.18	6	6	1	5.10	449.54	1
**9b**	84.09	72.18	6	6	1	5.91	528.43	2
**12**	66.87	122.11	5	9	2	4.37	518.56	1
**16b**	75.02	98.48	7	8	1	4.66	507.57	1
**17**	81.42	79.93	5	7	0	4.24	465.54	0
**Celecoxib**	82.09	77.99	4	5	2	3.61	381.38	0
**Indomethacin**	85.35	68.54	4	5	1	3.99	357.79	0

## Supplementary Materials

The Supplementary Materials are available online.

## References

[1] Chen L., Deng H., Cui H., Fang J., Zuo Z., Deng J., Li Y., Wang X., Zhao L. (2017). Inflammatory responses and inflammation-associated diseases in organs. Oncotarget.

[2] Varela M.L., Mogildea M., Moreno I., Lopes A. (2018). Acute Inflammation and Metabolism. Inflammation.

[3] Nasef N.A., Mehta S., Ferguson L.R. (2017). Susceptibility to chronic inflammation: An update. Arch. Toxicol..

[4] Al Rashoud A.S., Abboud R.J., Wang W., Wigderowitz C. (2014). Efficacy of low-level laser therapy applied at acupuncture points in knee osteoarthritis: A randomised double-blind comparative trial. Physiotherapy.

[5] Lee A.S., Ellman M.B., Yan D., Kroin J.S., Cole B.J., Van Wijnen A.J., Im H.J. (2013). A current review of molecular mechanisms regarding osteoarthritis and pain. Gene.

[6] Sehajpal S., Prasad D.N., Singh R.K. (2018). Prodrugs of Non-steroidal Anti-inflammatory Drugs (NSAIDs): A Long March Towards Synthesis of Safer NSAIDs. Mini Rev. Med. Chem..

[7] Radi Z.A., Khan K.N. (2019). Cardio-renal safety of non-steroidal anti-inflammatory drugs. J. Toxicol. Sci..

[8] Korbecki J., Baranowska-Bosiacka I., Gutowska I., Chlubek D. (2014). Cyclooxygenase pathways. Acta Biochim. Pol..

[9] Tortorella M.D., Zhang Y., Talley J. (2016). Desirable Properties for 3rd Generation Cyclooxygenase-2 Inhibitors. Mini Rev. Med. Chem..

[10] Kumar V., Kaur K., Gupta G.K., Gupta A.K., Kumar S. (2013). Developments in synthesis of the anti-inflammatory drug, celecoxib: A review. Recent Pat. Inflamm. Allergy Drug Discov..

[11] Martina S.D., Vesta K.S., Ripley T.L. (2005). Etoricoxib: A highly selective COX-2 inhibitor. Ann. Pharmacother..

[12] Burnier M. (2005). The safety of rofecoxib. Expert Opin. Drug Saf..

[13] Fanelli A., Ghisi D., Aprile P.L., Lapi F. (2017). Cardiovascular and cerebrovascular risk with nonsteroidal anti-inflammatory drugs and cyclooxygenase 2 inhibitors: Latest evidence and clinical implications. Ther. Adv. Drug Saf..

[14] Carullo G., Galligano F., Aiello F. (2016). Structure-activity relationships for the synthesis of selective cyclooxygenase 2 inhibitors: An overview (2009–2016). MedChemComm.

[15] Khalil N.A., Ahmed E.M., Mohamed K.O., Nissan Y.M., Zaitone S.A. (2014). Synthesis and biological evaluation of new pyrazolone-pyridazine conjugates as anti-inflammatory and analgesic agents. Bioorg. Med. Chem..

[16] Saeed M.M., Khalil N.A., Ahmed E.M., Eissa K.I. (2012). Synthesis and anti-inflammatory activity of novel pyridazine and pyridazinone derivatives as non-ulcerogenic agents. Arch. Pharm. Res..

[17] Bingham S., Beswick P.J., Bountra C., Brown T., Campbell I.B., Chessell I.P., Clayton N., Collins S.D., Davey P.T., Goodland H. (2005). The cyclooxygenase-2 inhibitor GW406381X [2-(4-ethoxyphenyl)-3-[4-(methylsulfonyl)phenyl]-pyrazolo[1,5-b]pyridazine] is effective in animal models of neuropathic pain and central sensitization. J. Pharmacol. Exp. Ther..

[18] Ahmed E.M., Kassab A.E., El-Malah A.A., Hassan M.S.A. (2019). Synthesis and biological evaluation of pyridazinone derivatives as selective COX-2 inhibitors and potential anti-inflammatory agents. Eur. J. Med. Chem..

[19] Singh J., Sharma D., Bansal R. (2017). Pyridazinone: An attractive lead for anti-inflammatory and analgesic drug discovery. Future Med. Chem..

[20] Luong C., Miller A., Barnett J., Chow J., Ramesha C., Browner M.F. (1996). Flexibility of the NSAID binding site in the structure of human cyclooxygenase-2. Nat. Struct. Biol..

[21] Fabiola G., Damodharan L., Pattabhi V., Nagarajan K. (2001). Cyclooxygenase-2—An attractive target for fruitful drug design. Curr. Sci..

[22] Liaras K., Fesatidou M., Geronikaki A. (2018). Thiazoles and Thiazolidinones as COX/LOX Inhibitors. Molecules.

[23] Abdellatif K.R., Abdelgawad M.A., Elshemy H.A., Alsayed S.S. (2016). Design, synthesis and biological screening of new 4-thiazolidinone derivatives with promising COX-2 selectivity, anti-inflammatory activity and gastric safety profile. Bioorg. Chem..

[24] Kaminskyy D., Kryshchyshyn A., Lesyk R. (2017). 5-Ene-4-thiazolidinones—An efficient tool in medicinal chemistry. Eur. J. Med. Chem..

[25] Omar Y.M., Abdu-Allah H.H.M., Abdel-Moty S.G. (2018). Synthesis, biological evaluation and docking study of 1,3,4-thiadiazole-thiazolidinone hybrids as anti-inflammatory agents with dual inhibition of COX-2 and 15-LOX. Bioorg. Chem..

[26] Tripathi A.C., Gupta S.J., Fatima G.N., Sonar P.K., Verma A., Saraf S.K. (2014). 4-Thiazolidinones: The advances continue. Eur. J. Med. Chem..

[27] Unsal-Tan O., Ozadali K., Piskin K., Balkan A. (2012). Molecular modeling, synthesis and screening of some new 4-thiazolidinone derivatives with promising selective COX-2 inhibitory activity. Eur. J. Med. Chem..

[28] Verma A., Saraf S.K. (2008). 4-thiazolidinone—A biologically activescaffold. Eur. J. Med. Chem..

[29] El-Feky S.A., Imran M., Nayeem N. (2017). Design, Synthesis, and Anti-Inflammatory Activity of Novel Quinazolines. Orient. J. Chem..

[30] Khan A., Diwan A., Thabet H.K., Imran M. (2020). Synthesis of novel N-substitutedphenyl-6-oxo-3-phenyl pyridazine derivatives as cyclooxygenase-2 inhibitors. Drug Dev. Res..

[31] El-Feky S.A., Abd El-Samii Z.K., Osman N.A., Lashine J., Kamel M.A., Thabet H.K. (2015). Synthesis, molecular docking and anti-inflammatory screening of novel quinoline incorporated pyrazole derivatives using the Pfitzinger reaction II. Bioorg. Chem..

[32] El-Feky S.A., Hamdy K.T., Mustafa T.U. (2014). Synthesis, molecular modeling and anti-inflammatory screening of novel fluorinated quinoline incorporated benzimidazole derivatives using the Pfitzinger reaction. J. Fluor. Chem..

[33] Matheus M.E., ViolanteFde A., Garden S.J., Pinto A.C., Fernandes P.D. (2007). Isatins inhibit cyclooxygenase-2 and inducible nitric oxide synthase in a mouse macrophage cell line. Eur. J. Pharmacol..

[34] Winter C.A., Risley E.A., Nuss G.W. (1962). Carrageenin-induced edema in hind paw of the rat as an assay for antiiflammatory drugs. Proc. Soc. Exp. Biol. Med..

[35] Ohkawa H., Ohishi N., Yagi K. (1979). Assay for lipid peroxides in animaltissues by thiobarbituric acid reaction. Anal. Biochem..

[36] Banerjee A.G., Das N., Shengule S.A., Sharma P.A., Srivastava R.S., Shrivastava S.K. (2016). Design, synthesis, evaluation and molecular modelling studies of some novel 5,6-diphenyl-1,2,4-triazin-3(2H)-ones bearing five-member heterocyclic moieties as potential COX-2 inhibitors: A hybrid pharmacophore approach. Bioorg. Chem..

[37] Orlando B.J., Malkowski M.G. (2016). Crystal structure of rofecoxib bound to human cyclooxygenase-2. Acta Crystallogr. F Struct. Biol. Commun..

[38] Pérez-Villanueva J., Yépez-Mulia L., González-Sánchez I., Palacios-Espinosa J.F., Soria-Arteche O., Sainz-Espuñes T.D.R., Cerbón M.A., Rodríguez-Villar K., Rodríguez-Vicente A.K., Cortés-Gines M. (2017). Synthesis and Biological Evaluation of 2H-Indazole Derivatives: Towards Antimicrobial and Anti-Inflammatory Dual Agents. Molecules.

[39] Jiao J., Yang Y., Wu Z., Li B., Zheng Q., Wei S., Wang Y., Yang M. (2019). Screening cyclooxygenase-2 inhibitors from Andrographis paniculata to treat inflammation based on bio-affinity ultrafiltration coupled with UPLC-Q-TOF-MS. Fitoterapia.

[40] Molinspiration Cheminformatics. www.molinspiration.com/services/properties.html.

[41] Lipinski C.A., Lombardo L., Dominy B.W., Feeney P.J. (2001). Experimental and computational approaches to estime solubility and permeability in drug discovery and development settings. Adv. Drug Deliv. Rev..

[42] Benet L.Z., Hosey C.M., Ursu O., Oprea T.I. (2016). BDDCS, the Rule of 5 and drugability. Adv. Drug Deliv. Rev..

[43] Shakeel F., Imran M., Haq N., Alshehri S., Anwer M.K. (2019). Synthesis, Characterization and Solubility Determination of 6-Phenyl-pyridazin-3(2H)-one in Different Pharmaceutical Solvents. Molecules.

[44] Amin N.H., Mohammed A.A., Abdellatif K.R. (2018). Novel4-methylsulfonylphenylderivatives as NSAIDS with preferentialCOX-2inhibition. Future Med. Chem..

[45] Zhao Y., Abraham M.H., Lee J. (2002). Rate-limited steps of human oral absorption and QSAR studies. Pharm. Res..

