# Supplementary material for: Discovery of Novel Pyridazine-Based Cyclooxygenase-2 Inhibitors with a Promising Gastric Safety Profile
Source: Molecules. 2020 Apr 25;25(9):2002. doi: 10.3390/molecules25092002 (PMC7249006; doi:10.3390/molecules25092002)
Supplement: Supplementary file 1 [file molecules-25-02002-s001.pdf]

molecules

MDPI

Supplementary Data

Discovery of Novel Pyridazine Based Cyclooxygenase-2 Inhibitors With Promising Gastric Safety Profile

Abida Khan [1,*], Anupama Diwan [1], Hamdy K Thabet [2], Mohd Imran [3], and Md. Afroz Bakht [4]

[1] School of Pharmaceutical Sciences, Apeejay Stya University, Sohna - Palwal Road, Sohna – 122103, India
[2] Department of Chemistry, Faculty of Science, Northern Border University, Rafha 91911, PO Box 840, Saudi Arabia
[3] Department of Pharmaceutical Chemistry, Faculty of Pharmacy, Northern Border University, Rafha 91911, Saudi Arabia
[4] Department of Chemistry, College of Science and Humanities in Al-Kharj, Prince Sattam Bin Abdulaziz University, Al-Kharj 11942, Saudi Arabia
* Correspondence: aqua_abkhan@yahoo.com; Tel.: +91-8053027613

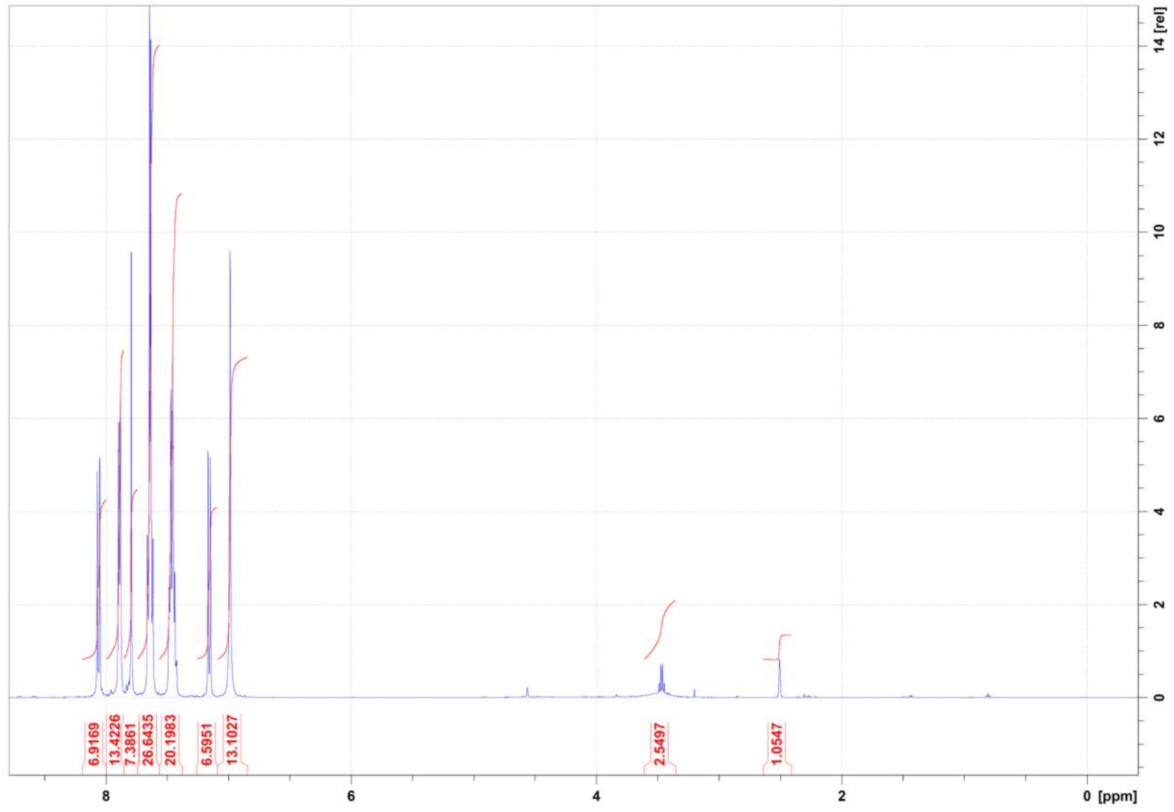

Figure 1. 1H-NMR spectrum of Compound 4a.

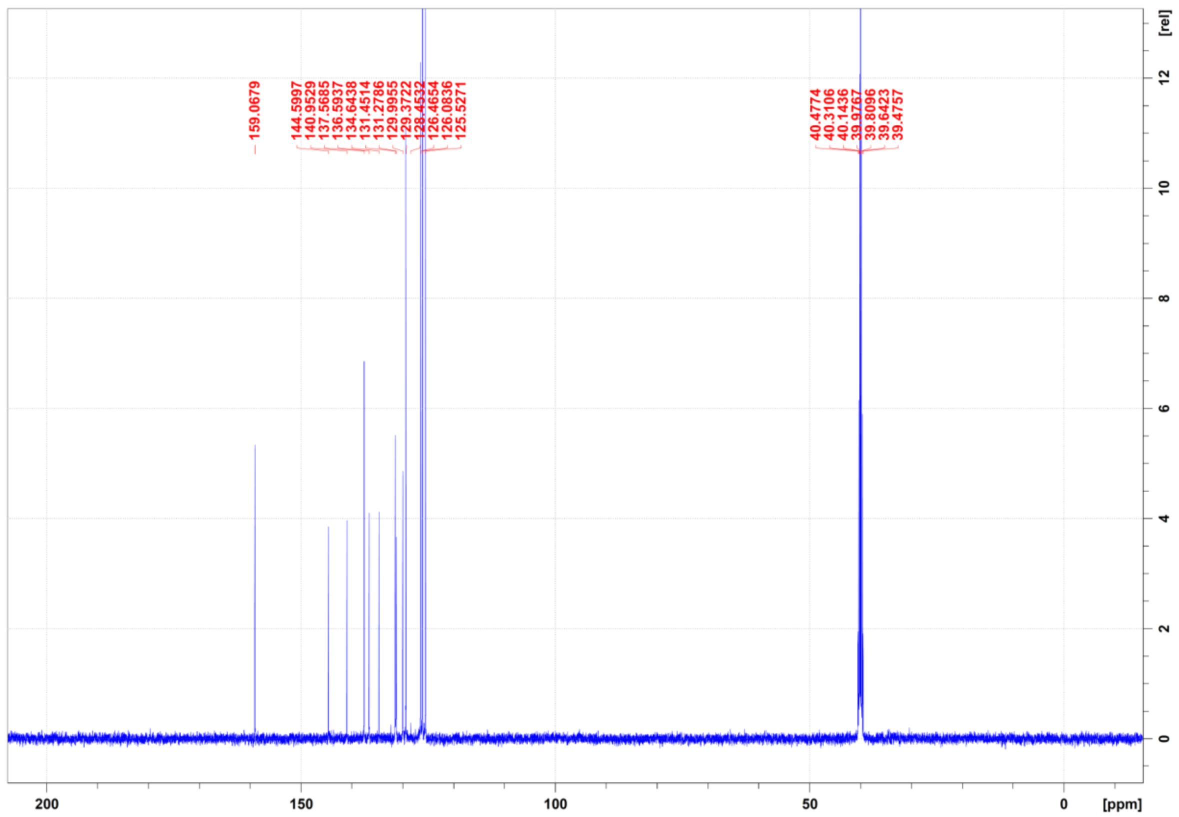

Figure 2. 13C-NMR spectrum of Compound 4a.

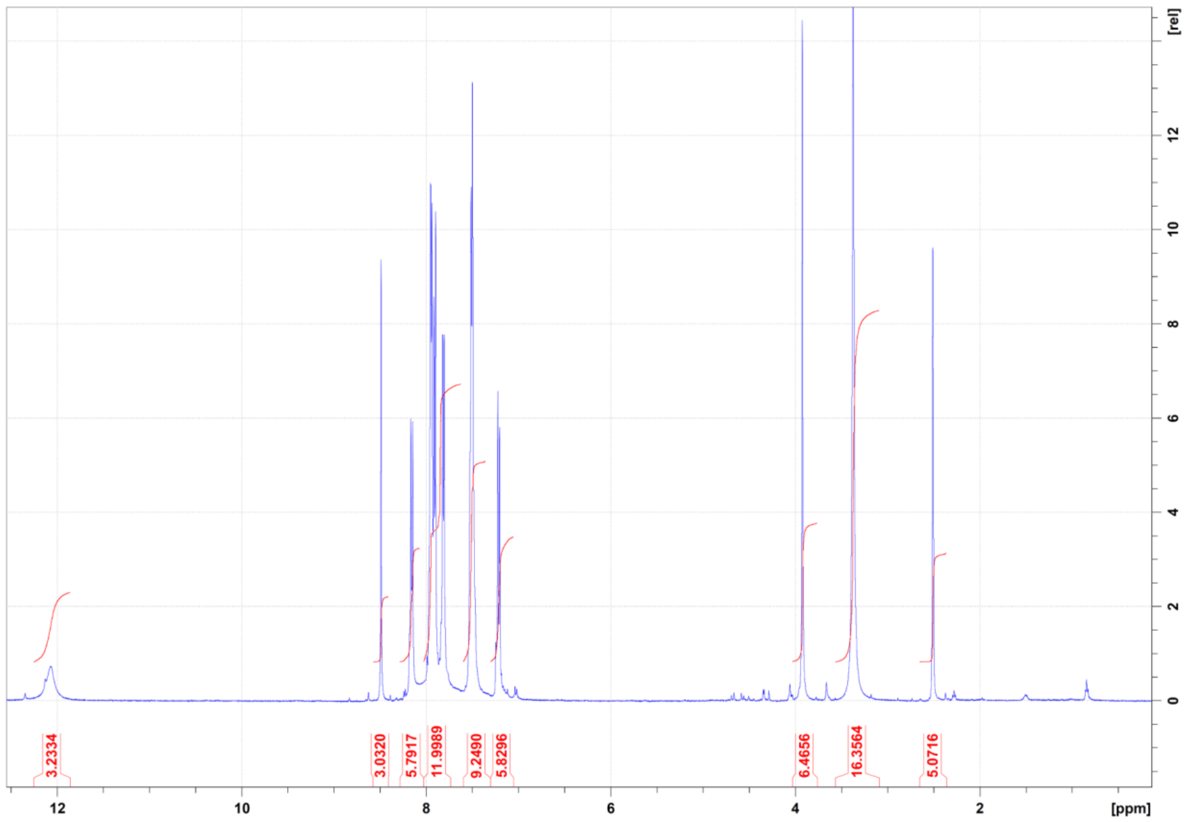

Figure 3. 1H-NMR spectrum of Compound 6a.

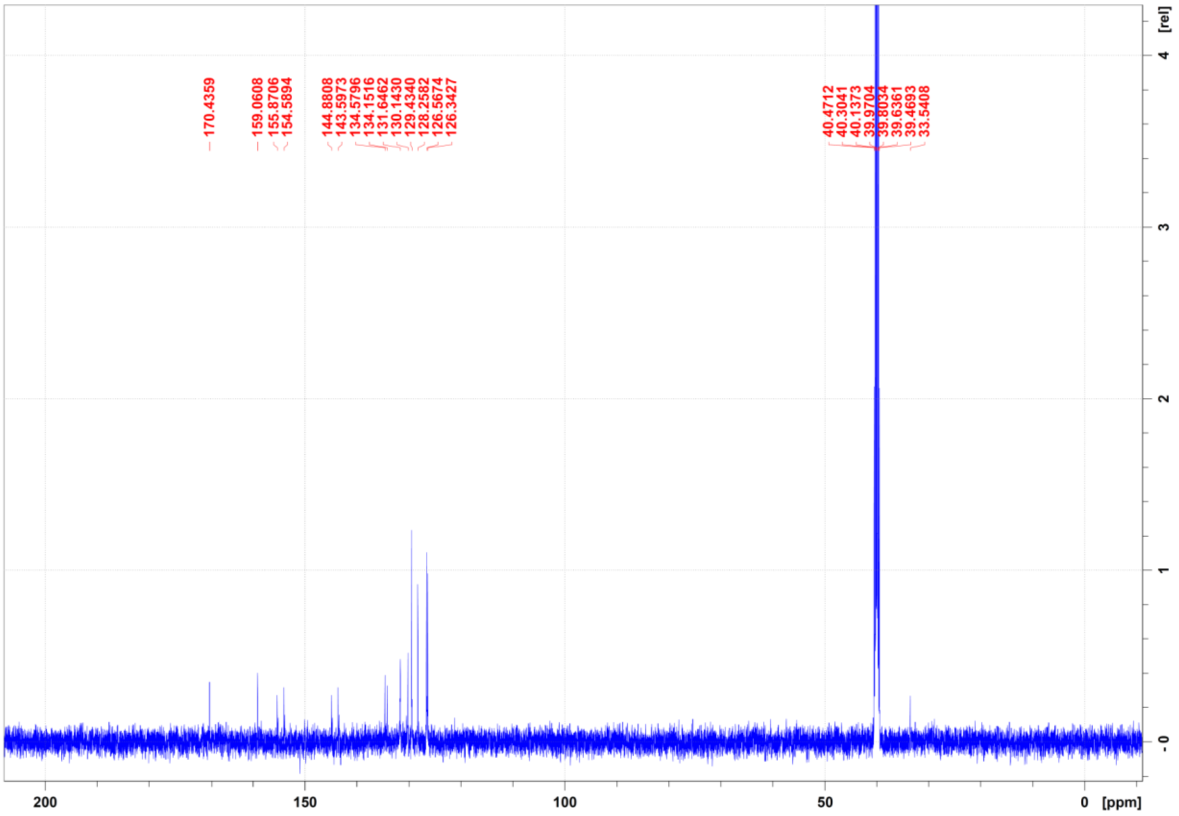

Figure 4. 13C-NMR spectrum of Compound 6a.

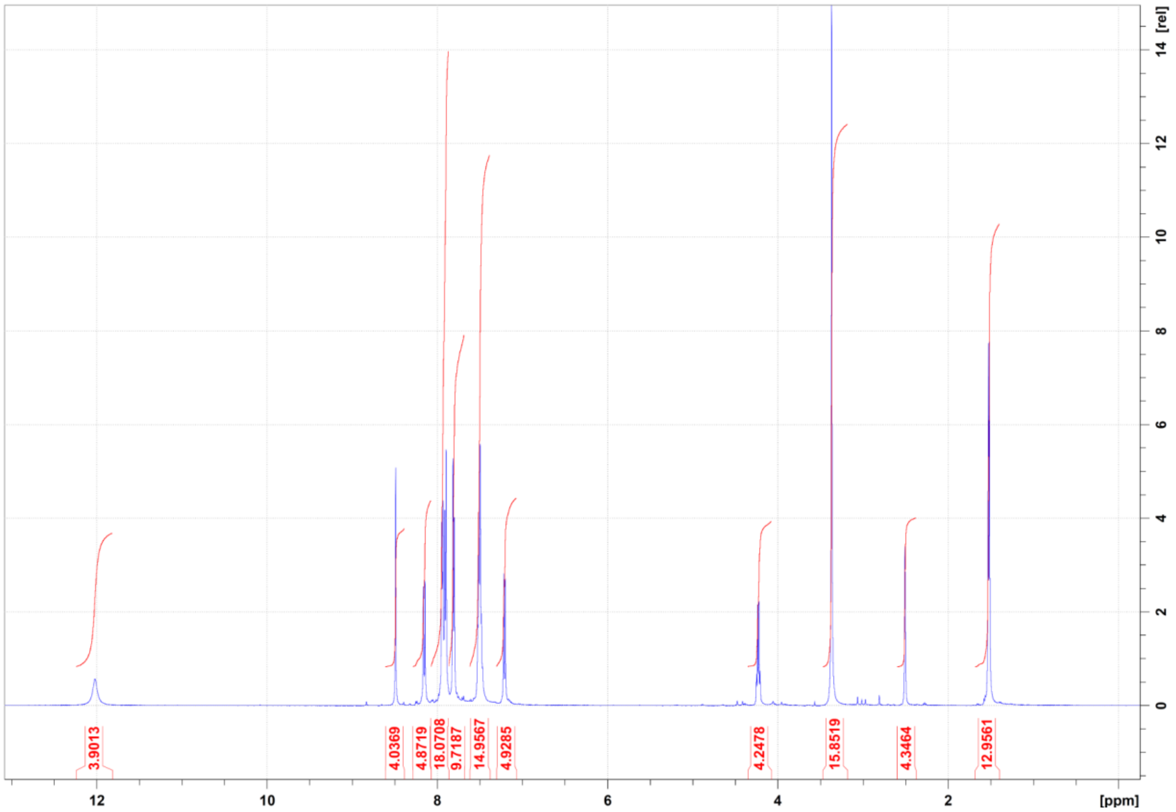

Figure 5. 1H-NMR spectrum of Compound 6b.

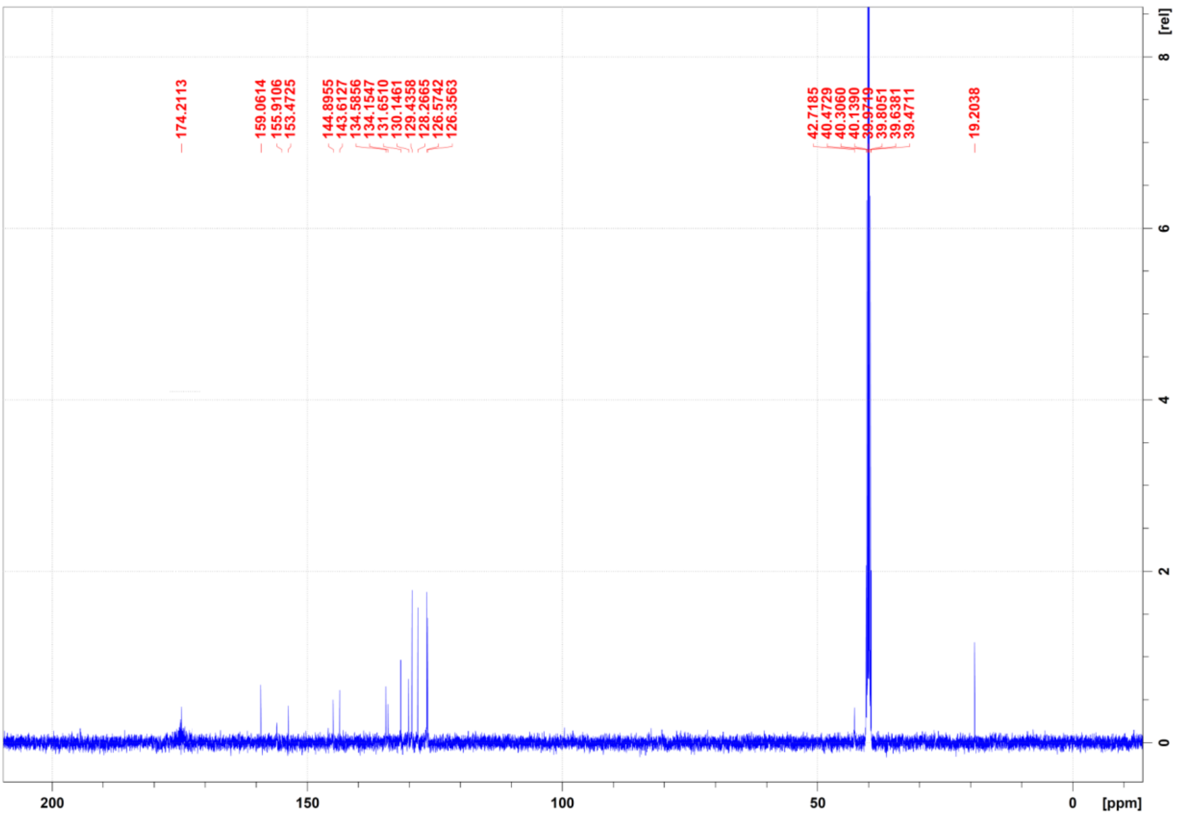

Figure 6. 13C-NMR spectrum of Compound 6b.

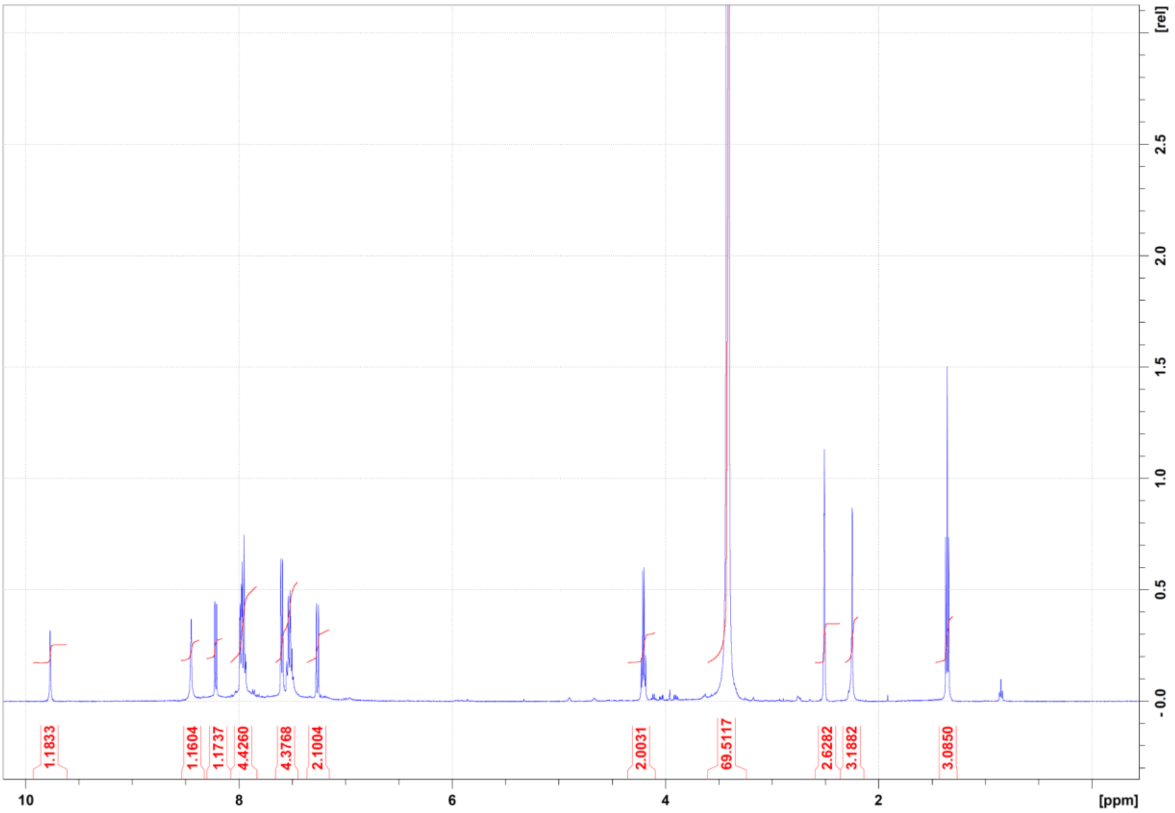

Figure 7. 1H-NMR spectrum of Compound 8.

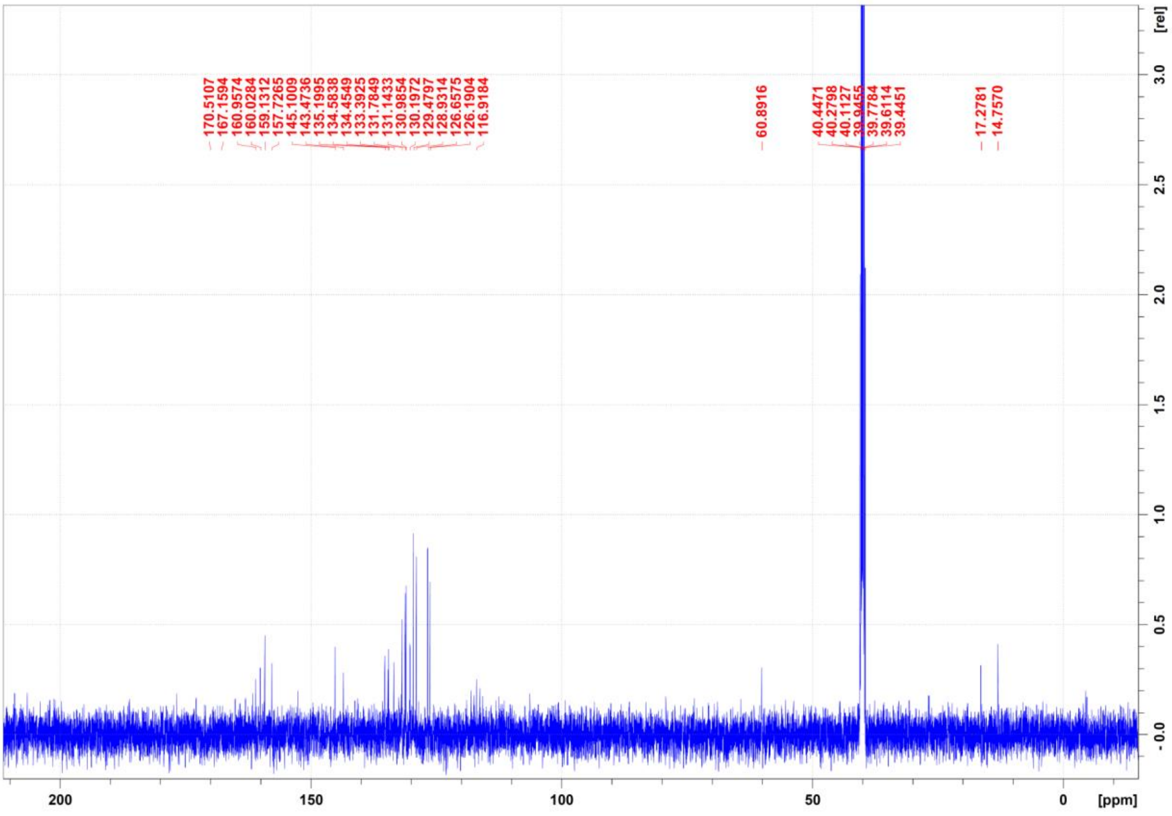

Figure 8. 13C-NMR spectrum of Compound 8.

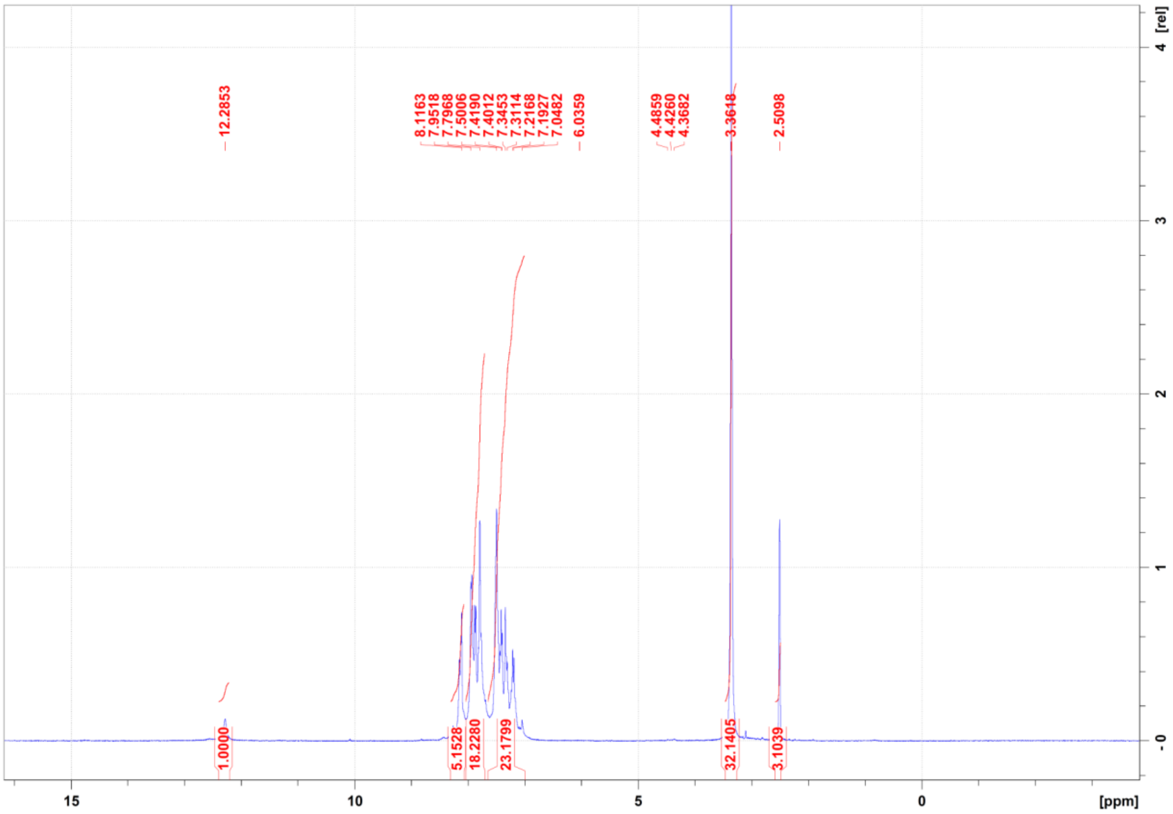

Figure 9. 1H-NMR spectrum of Compound 9a.

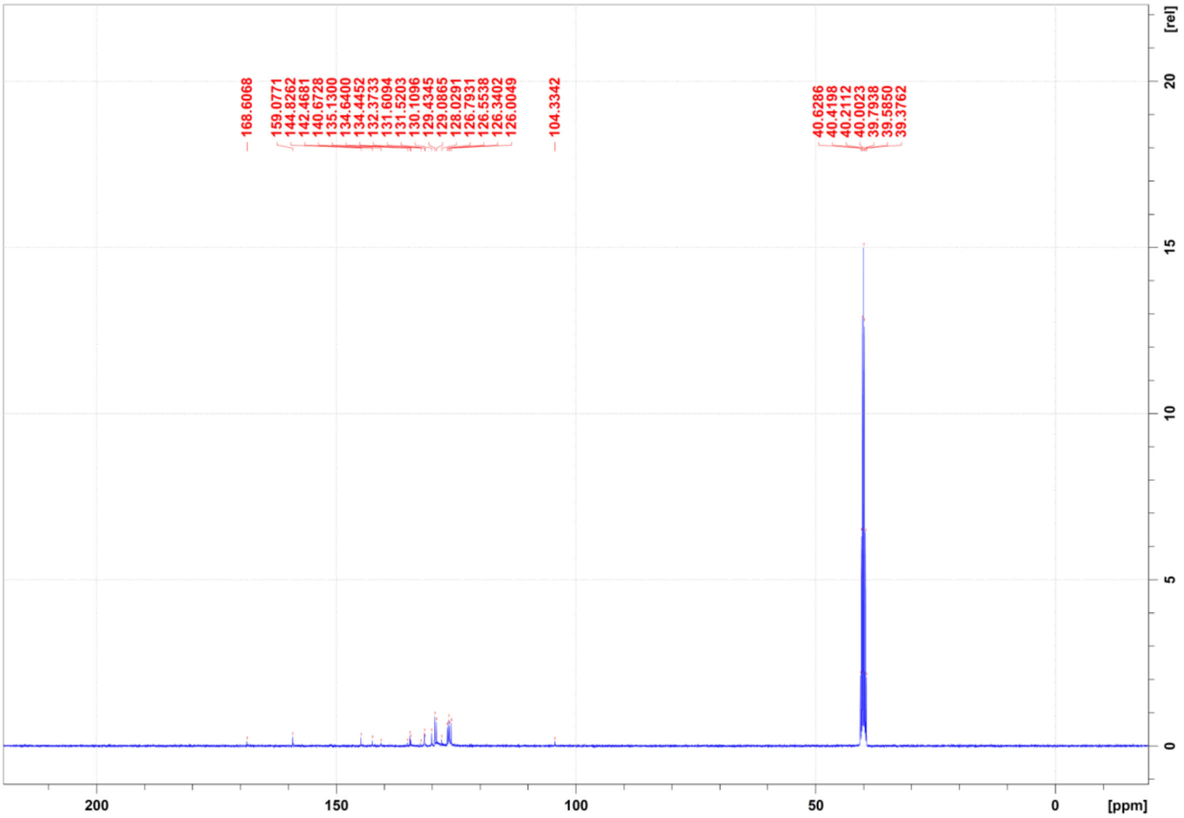

Figure 10. 13C-NMR spectrum of Compound 9a.

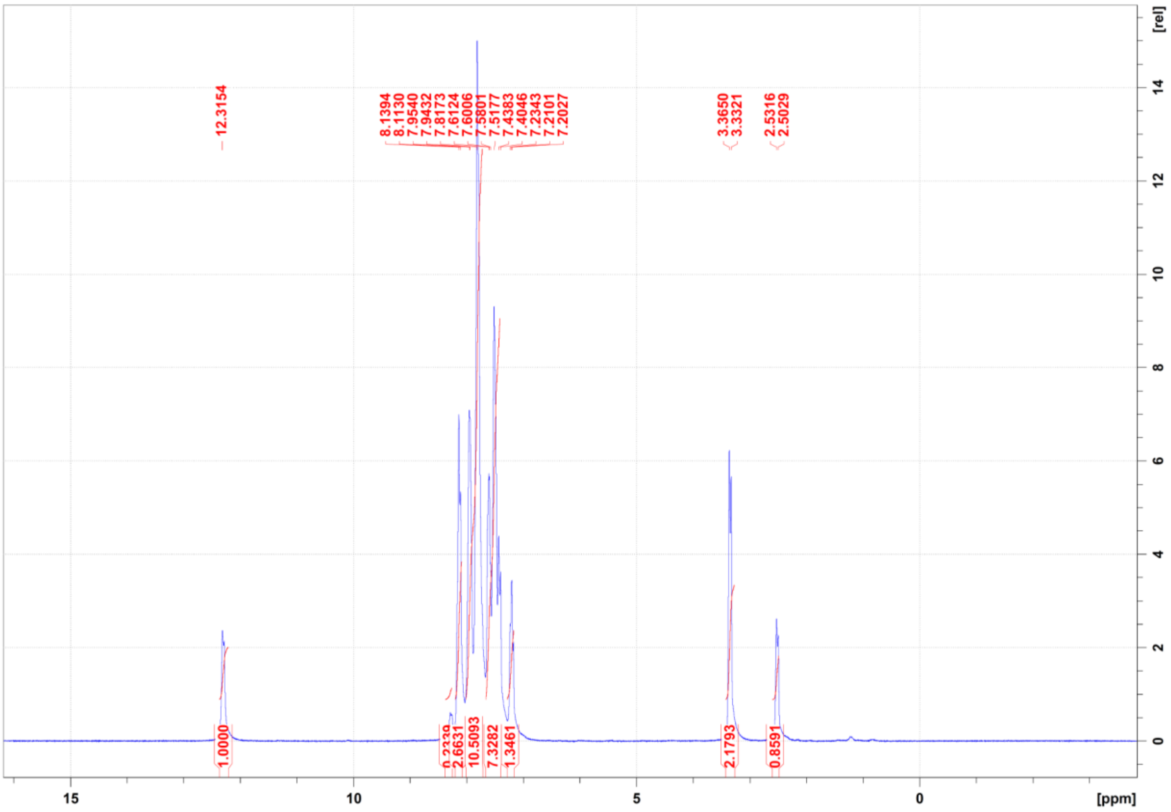

Figure 11. 1H-NMR spectrum of Compound 9b.

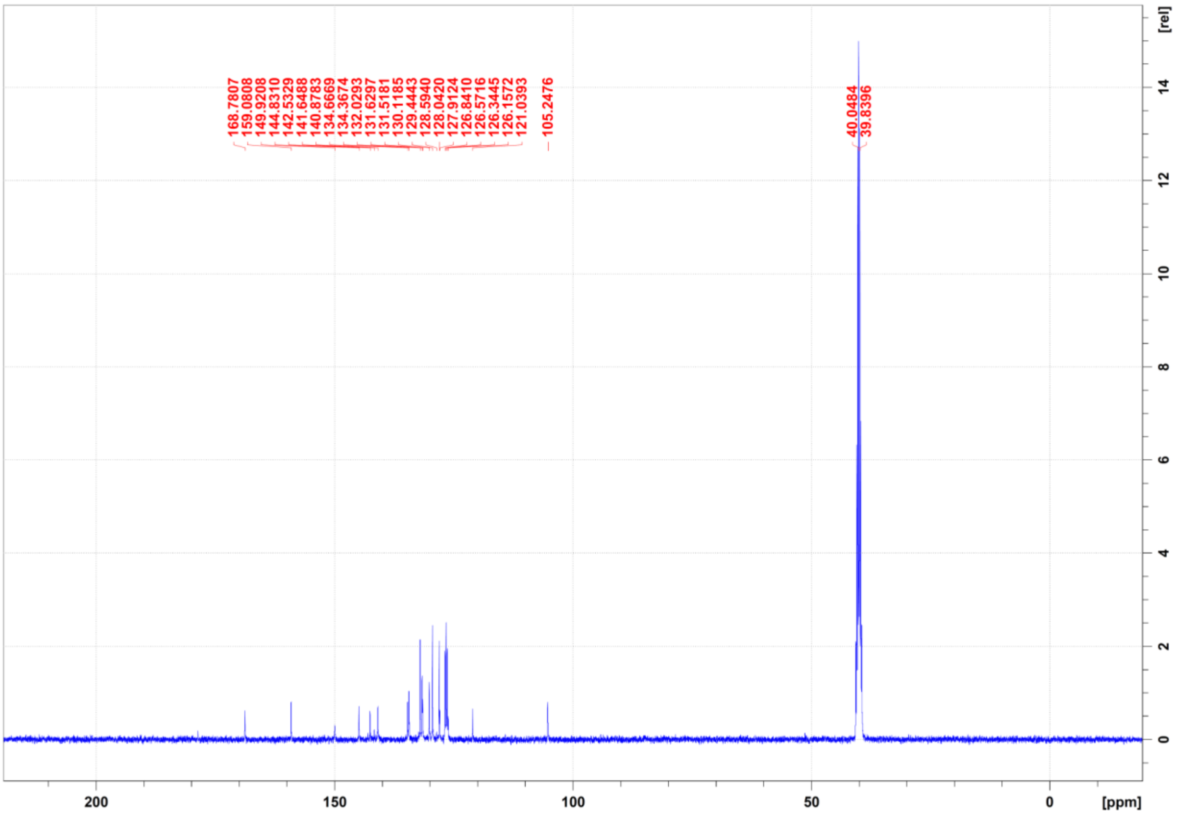

Figure 12. 13C-NMR spectrum of Compound 9b.

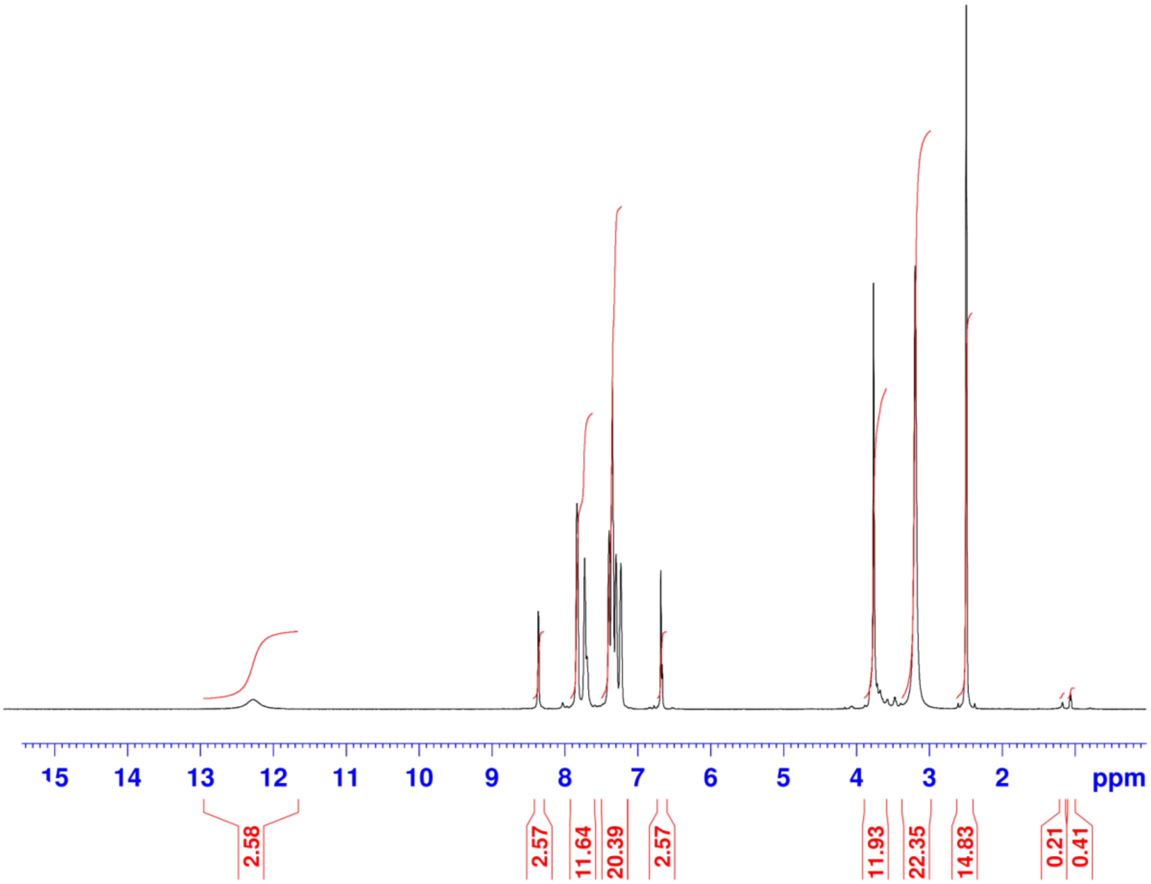

Figure 13. 1H-NMR spectrum of Compound 10.

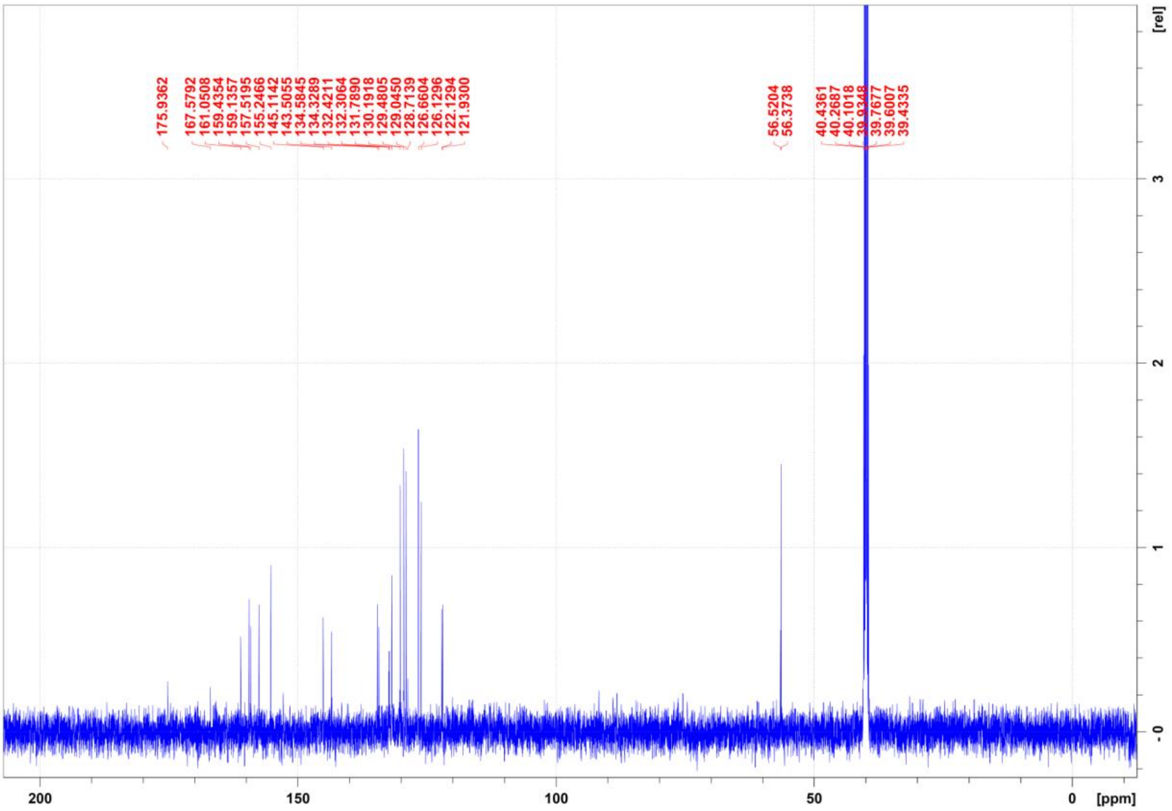

Figure 14. 13C-NMR spectrum of Compound 10.

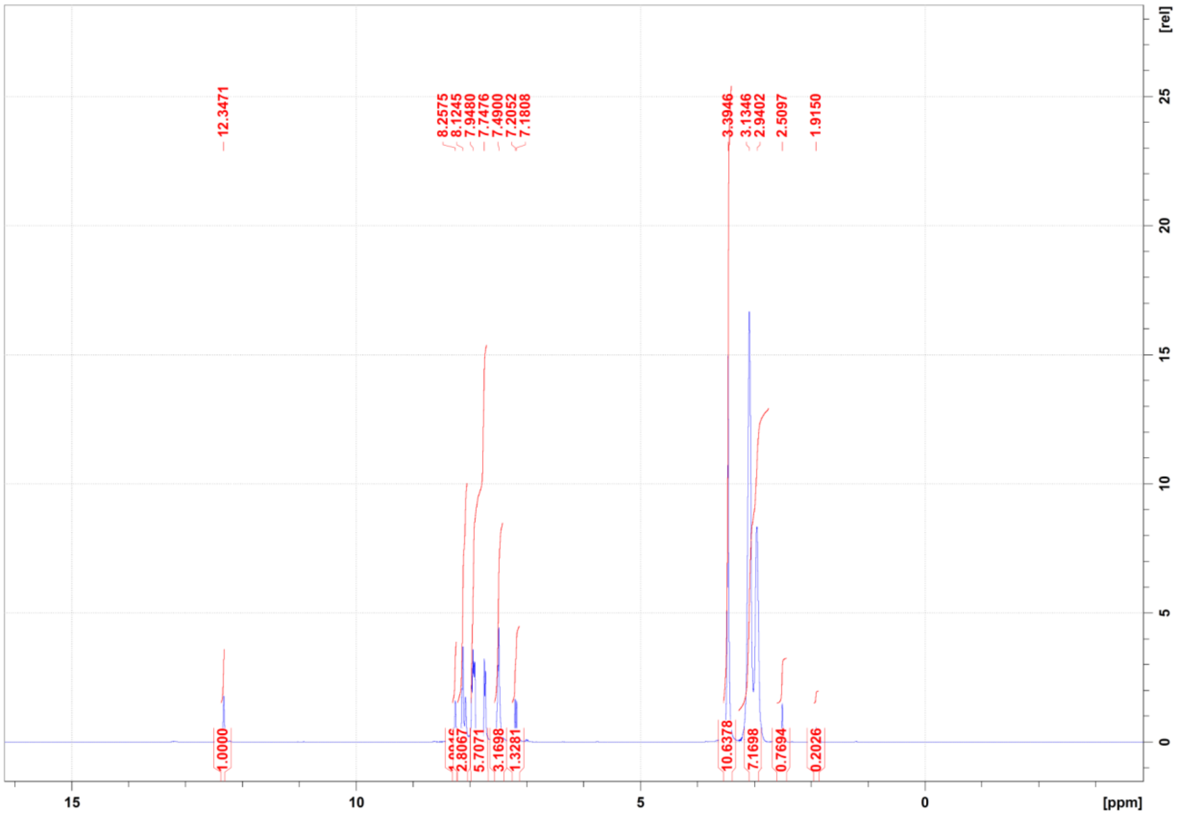

Figure 15. 1H-NMR spectrum of Compound 11.

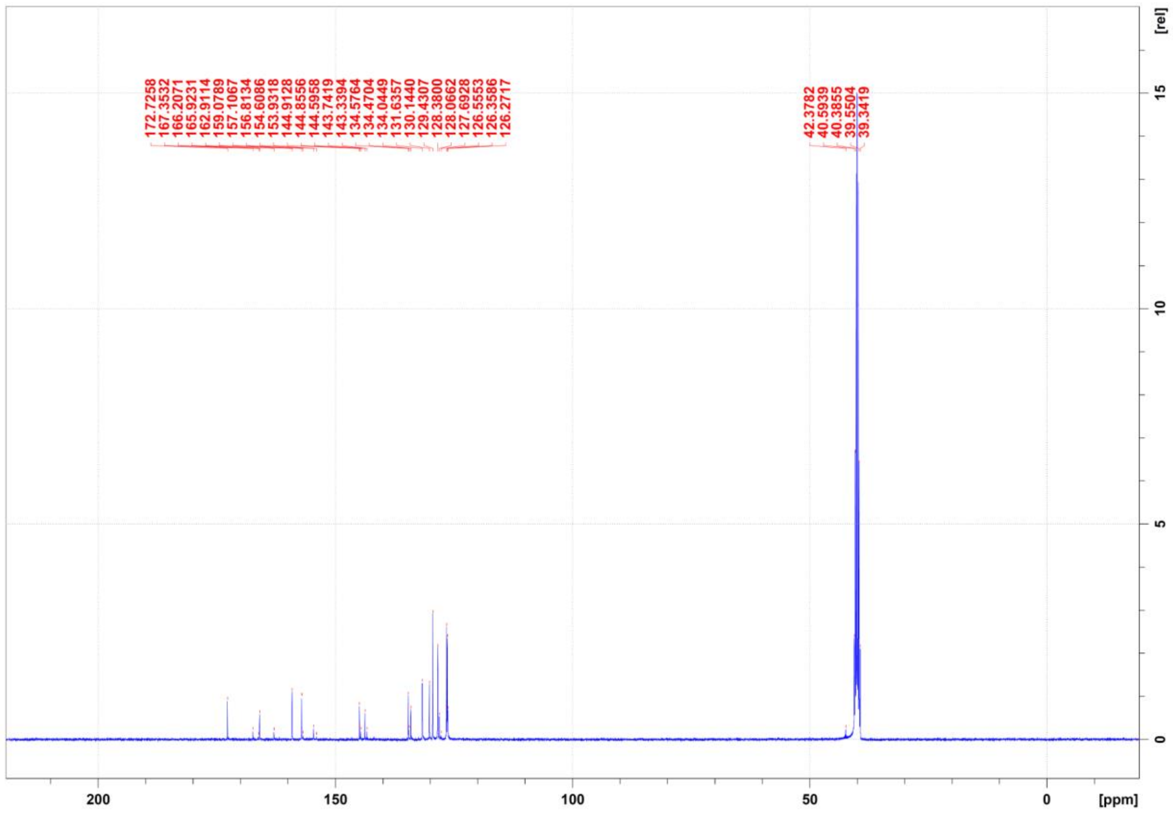

Figure 16. 13C-NMR spectrum of Compound 11.

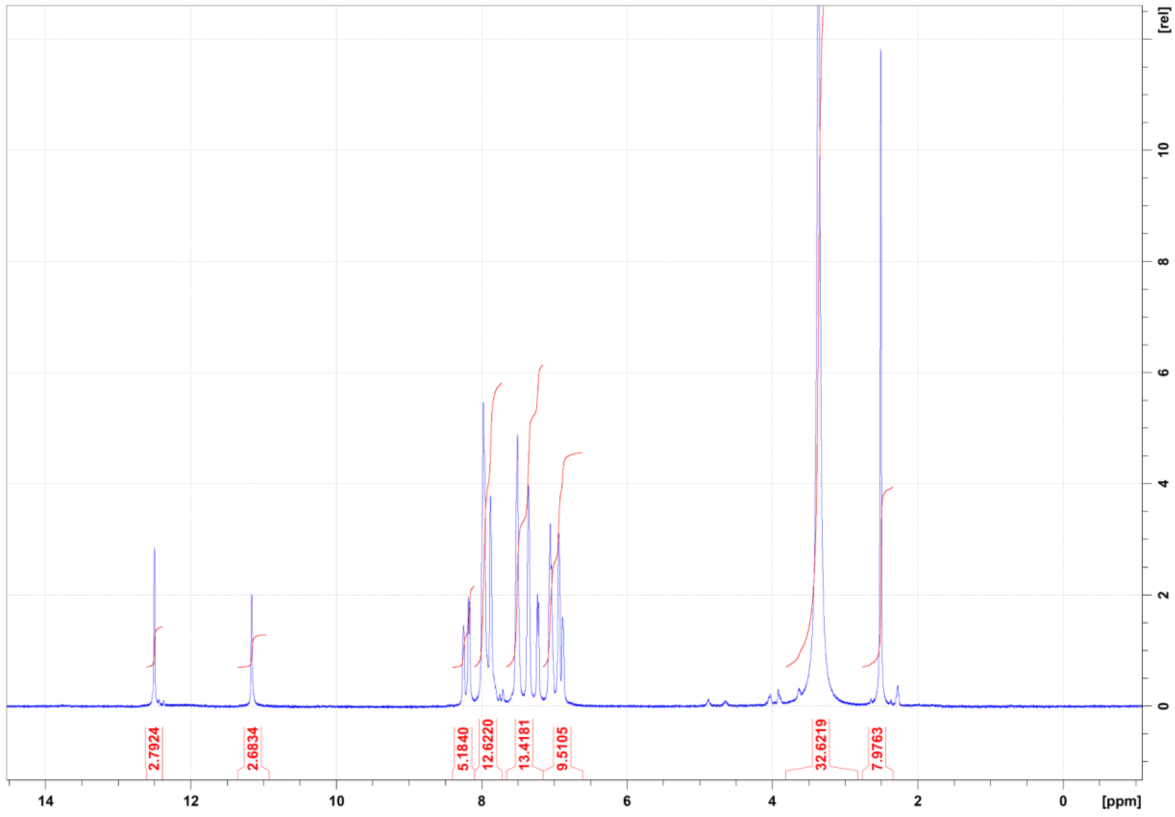

Figure 17. 1H-NMR spectrum of Compound 12.

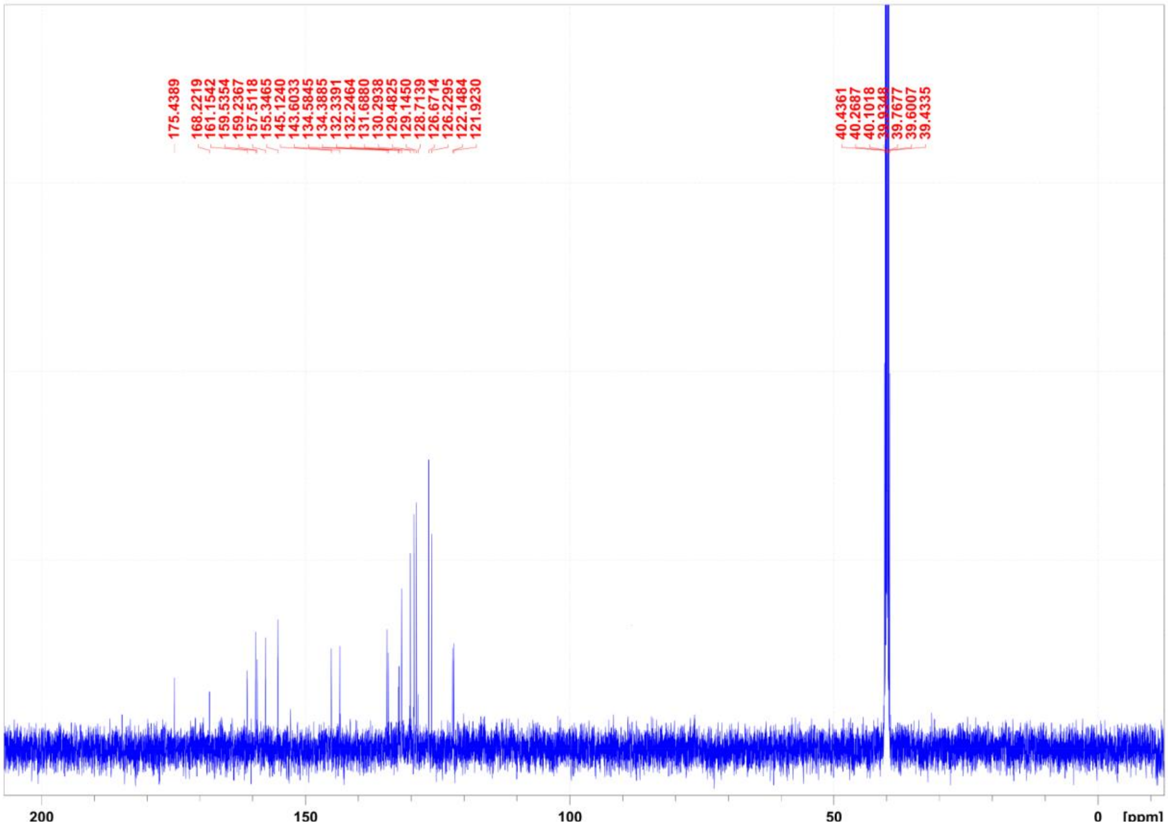

Figure 18. 13C-NMR spectrum of Compound 12.

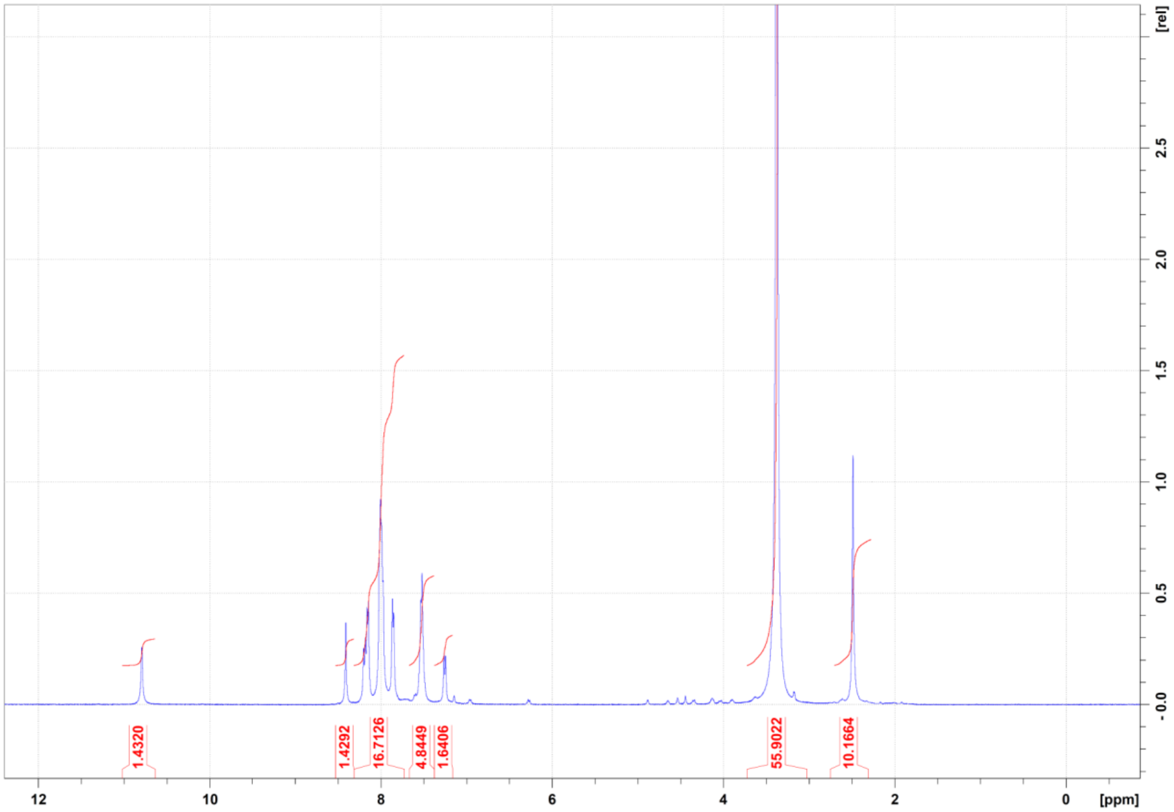

Figure 19. 1H-NMR spectrum of Compound 16a.

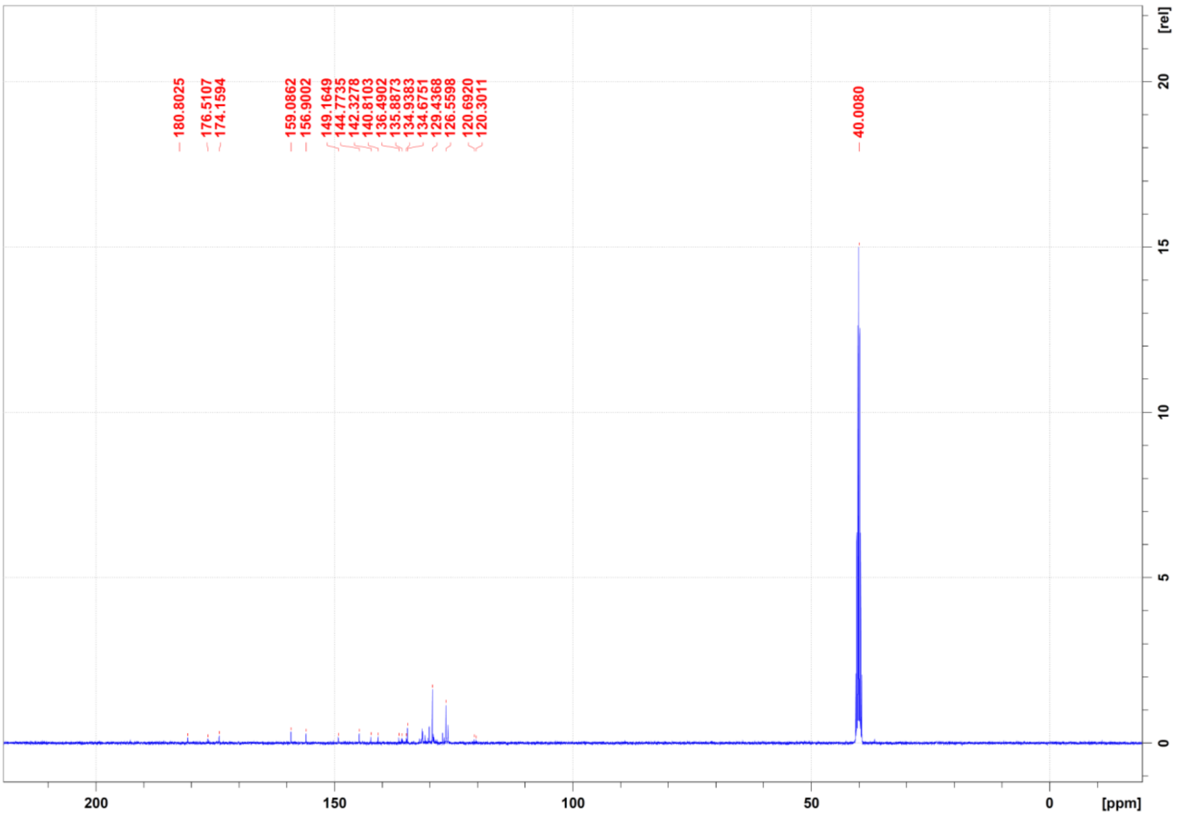

Figure 20. 13C-NMR spectrum of Compound 16a.

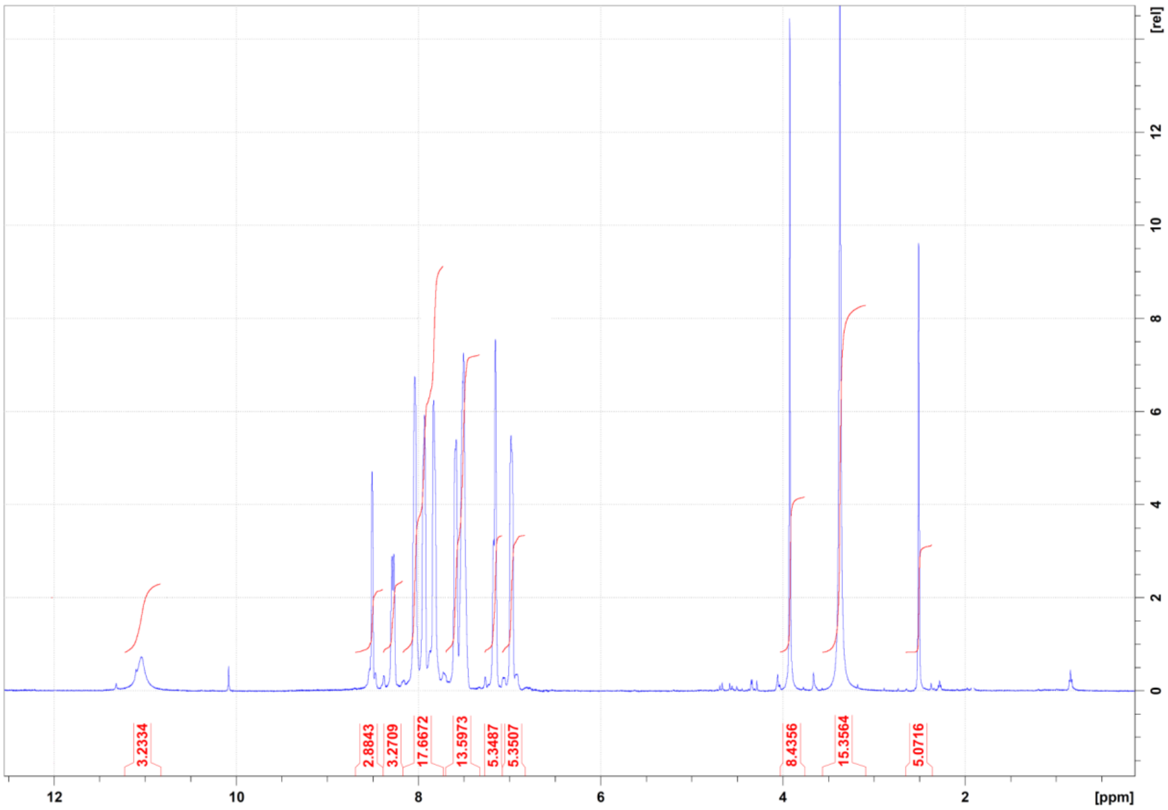

Figure 21. 1H-NMR spectrum of Compound 16b.

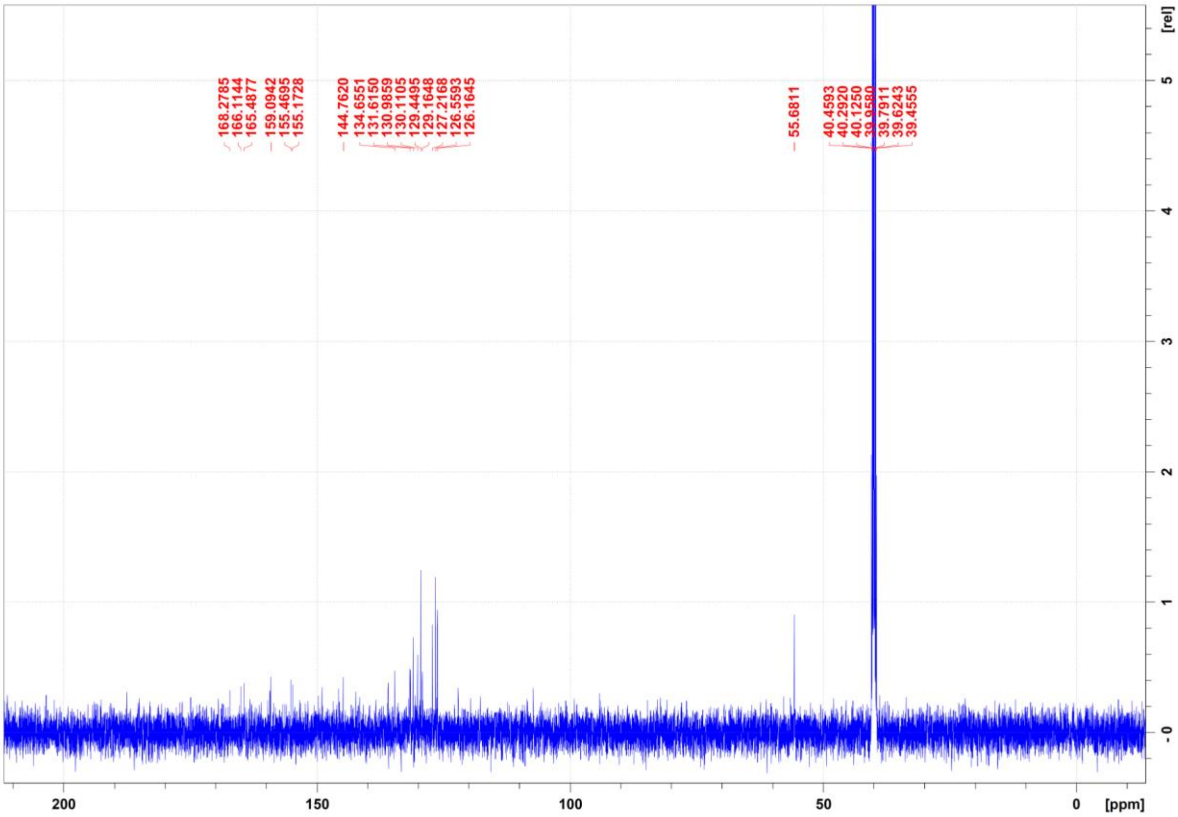

Figure 22. 13C-NMR spectrum of Compound 16b.

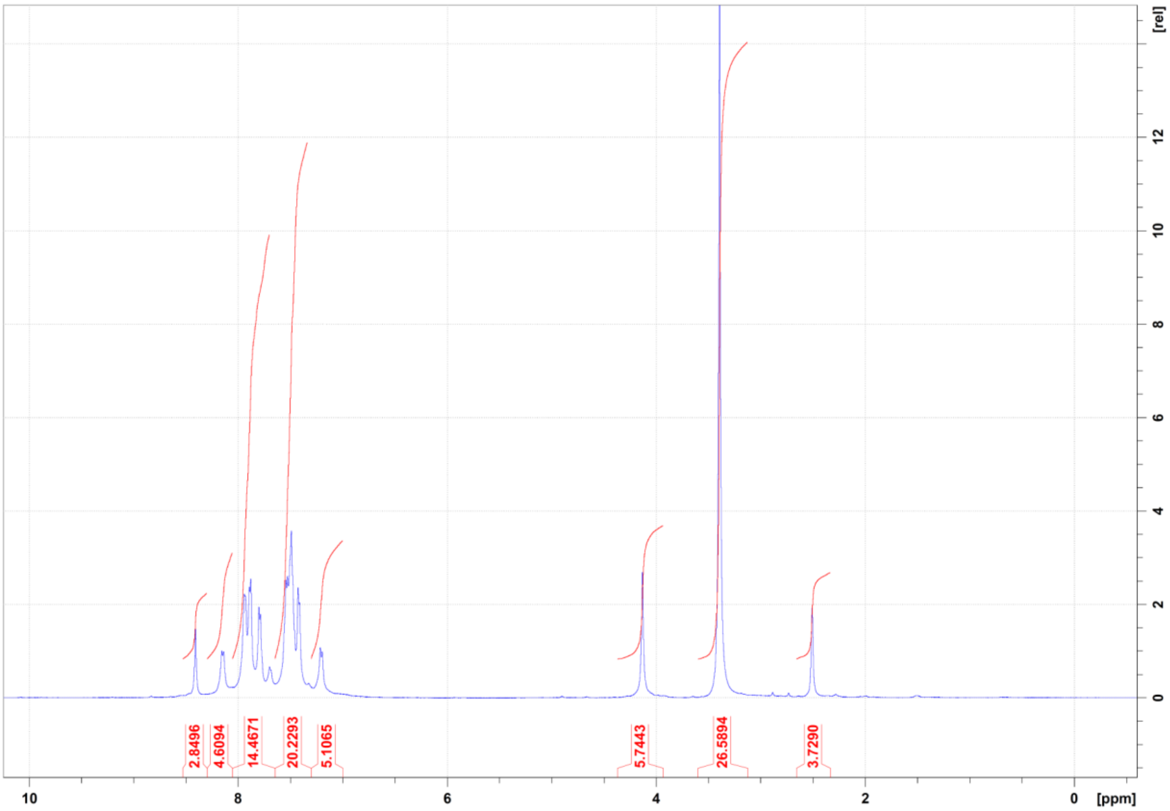

Figure 23. 1H-NMR spectrum of Compound 17.

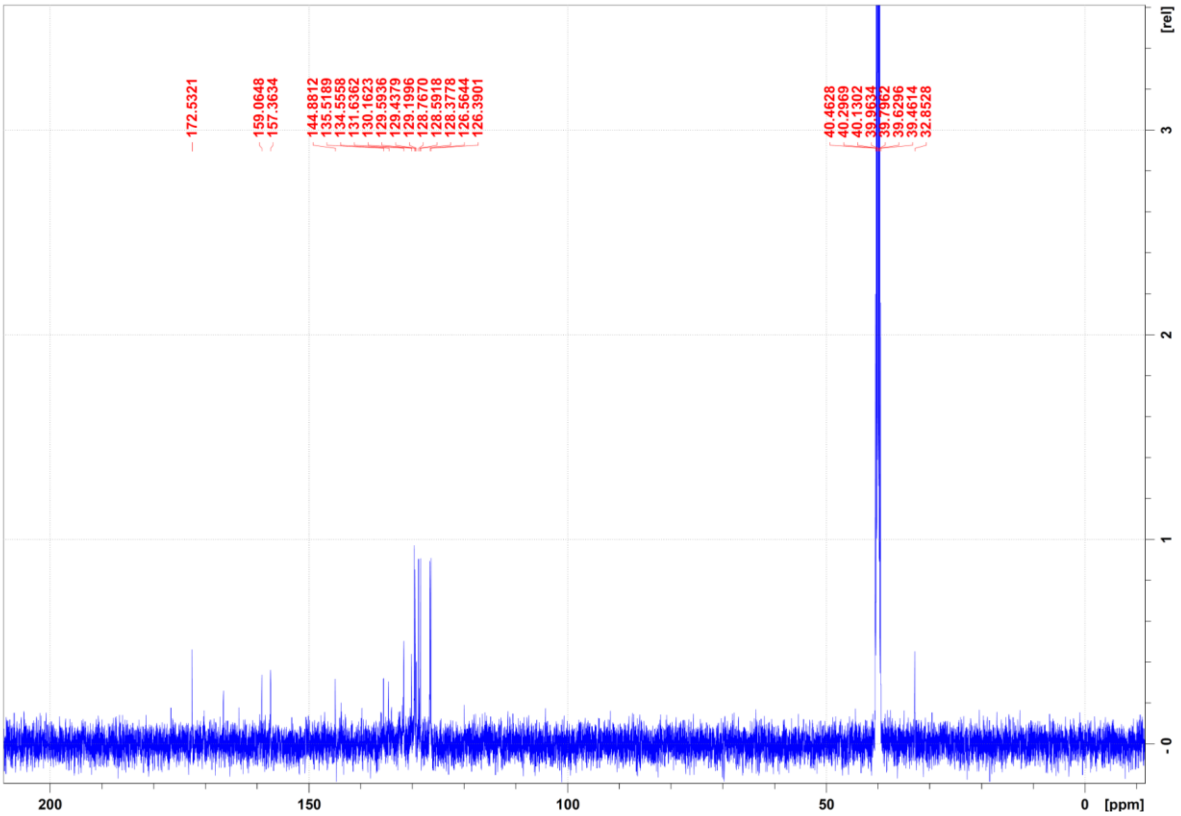

Figure 24. 13C-NMR spectrum of Compound 17.

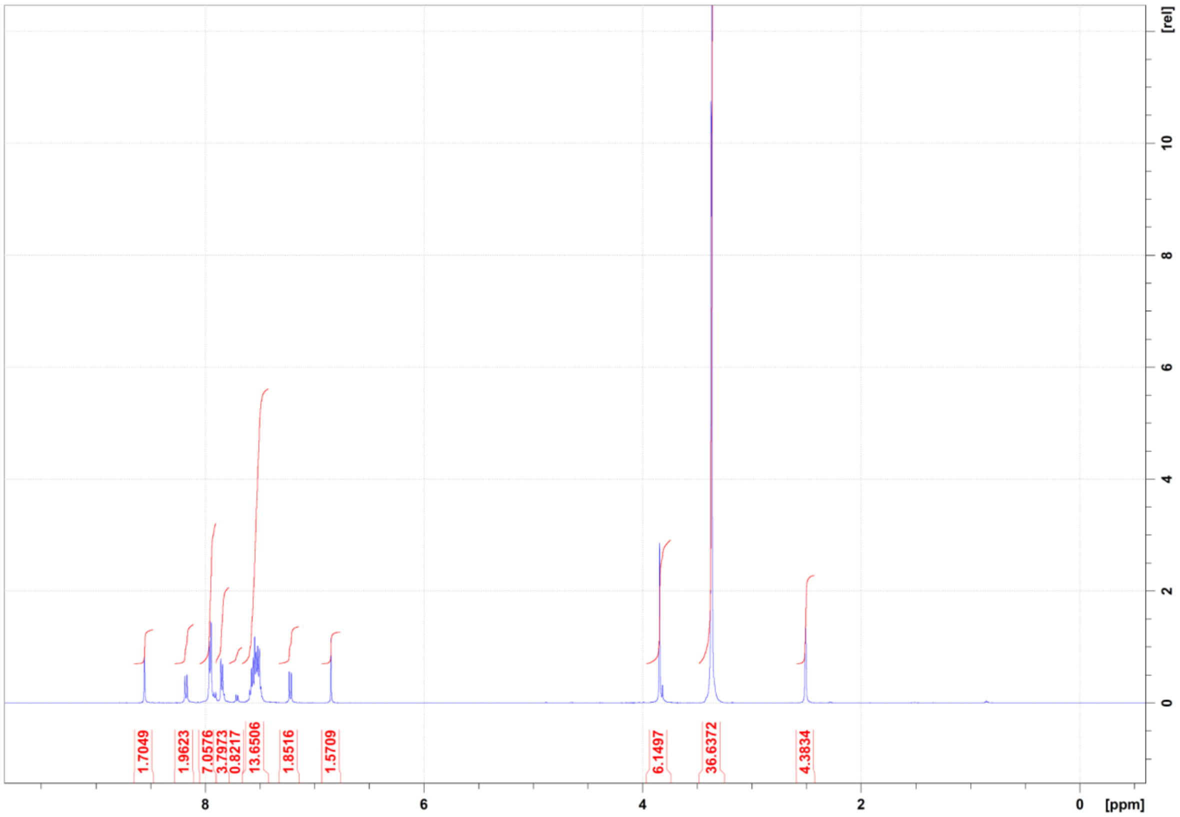

Figure 25. 1H-NMR spectrum of Compound 18.

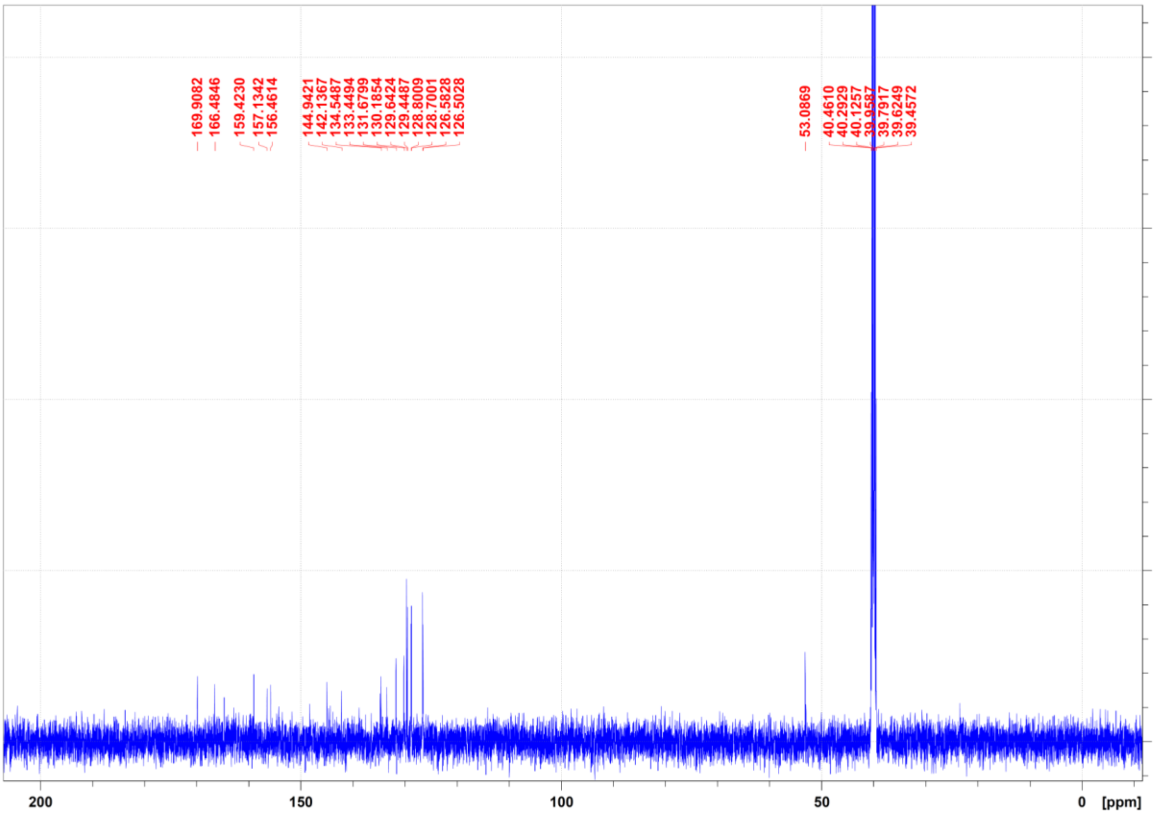

Figure 26. 13C-NMR spectrum of Compound 18.
